# Insights into the Spin Dynamics of Mononuclear Cerium(III) Single-Molecule Magnets

**DOI:** 10.1021/acs.inorgchem.2c00958

**Published:** 2022-07-11

**Authors:** Franz A. Mautner, Florian Bierbaumer, Roland C. Fischer, Ànnia Tubau, Saskia Speed, Eliseo Ruiz, Salah S. Massoud, Ramon Vicente, Silvia Gómez-Coca

**Affiliations:** † Institut für Physikalische und Theoretische Chemie, Technische Universität Graz, Stremayrgasse 9, A-8010 Graz, Austria; ‡ Institut für Anorganische Chemie, Technische Universität Graz, Stremayrgasse 9, A-8010 Graz, Austria; § Departament de Química Inorgànica i Orgànica, Universitat de Barcelona, Martí i Franquès 1-11, E-08028 Barcelona, Spain; ∥ Institut de Recerca de Química Teòrica i Computacional, Universitat de Barcelona, Martí i Franquès 1-11, E-08028 Barcelona, Spain; ⊥ Department of Chemistry, University of Louisiana at Lafayette, P.O. Box 43700, Lafayette, Louisiana 70504, United States

## Abstract

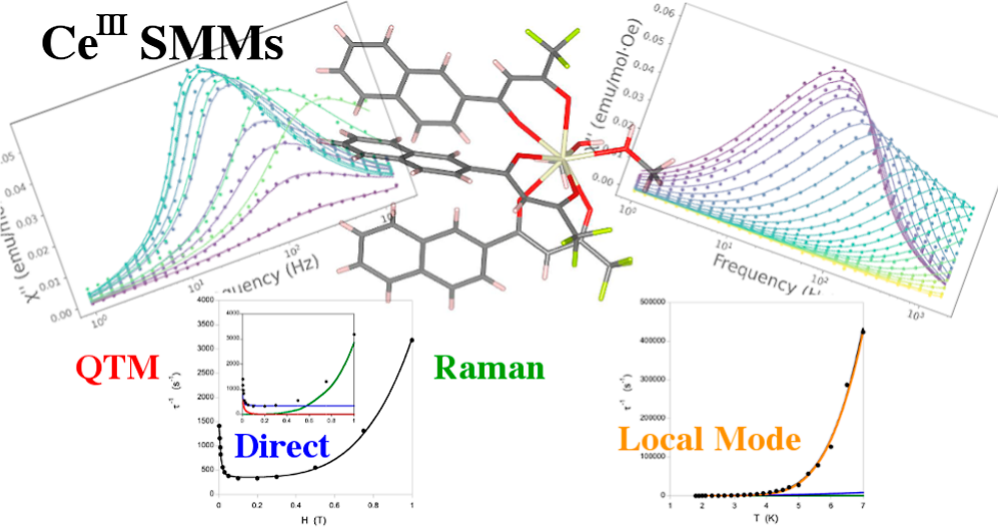

Four novel Ce^III^ mononuclear complexes of formulas [Ce(ntfa)_3_(MeOH)_2_] (**1**), [Ce(ntfa)_3_(5,5′-Me_2_bipy)] (**2**), [Ce(ntfa)_3_(terpy)] (**3**), and [Ce(ntfa)_3_(bipy)_2_] (**4**), where ntfa = 4,4,4-trifluoro-1-(naphthalen-2-yl)butane-1,3-dionato, 5,5′-Me_2_bipy = 5,5′-dimethyl-2,2′-dipyridyl, terpy = 2,2′:6′,2″-terpyridine, and bipy = 2,2′-bipyridine, have been synthesized and structurally characterized with Ce^III^ displaying coordination numbers of 8, 8, 9, and 10, respectively. Magnetic measurements indicate that all the complexes show a field-induced single-ion magnet behavior under a small applied dc field. The magnetic analysis shows the relevance of the different spin relaxation mechanisms in the magnetic relaxation of the Ce^III^ compounds, with special emphasis on the local-mode process. Multiconfigurational calculations were also performed to get more information on the axiality of the compounds.

## Abstract 2

Four novel Ce^III^ mononuclear complexes showing field-induced single-molecule magnet behavior at very low fields are presented. The Ce^III^ ions are coordinated by three β-diketone ligands, leaving other coordination positions to the solvent or polypyridyl ligands and showing the flexibility and tunability of the system. The importance of the different spin relaxation mechanisms in the magnetic relaxation of the Ce^III^ compounds is shown, specially the local-mode process.

## Introduction

Single-molecule magnets (SMMs) are molecules that have the capability to behave as small magnets.^1,2^ These molecules exhibit slow relaxation of the magnetization due to their intrinsic magnetic anisotropy, the property of the molecule that provides to its spin a preference to be oriented in a specific direction. Since their discovery,^3^ they have received great attention due to their possible application as data storage devices,^4^ in spintronics,^5,6^ and as spin qubits in quantum computing.^7−9^ Initially, the efforts were focused on transition metal compounds, until the discovery of a mononuclear Tb^III^ double-decker complex, [TbPc_2_]^−^·TBA^+^ [Pc = phthalocyanine and TBA^+^ = N(C_4_H_9_)_4_^+^], in 2003 by Ishikawa et al., which exhibited a SMM behavior.^10^ The isolation of this compound opened new avenues for the preparation of a plethora of mono and polynuclear SMM lanthanide (Ln) complexes with the aim of achieving high blocking temperatures (*T*_B_) and high energy barriers (*U*_eff_) for magnetization reversal.^11−16^

The large magnetic anisotropy usually observed in Ln^III^ metal ions is a result of the large unquenched orbital angular momentum and spin–orbit coupling.^17,18^ In contrast to what is usually observed in transition metal complexes, the spin–orbit coupling in lanthanide complexes is generally larger than the crystal field, and as a result, they are better described through the spin–orbit coupled term *J*. In the design of SMMs, Tb^III^, Dy^III^, and Er^III^ lanthanide ions were extensively used because of their large number of unpaired electrons (large angular momentum, *J* = *L* + *S*) and large magnetic anisotropy, which can also be enhanced with the appropriate choice of the ligand field, as proposed by Rinehart and Long.^19^ Indeed, a literature search on lanthanide-based SMMs shows that more than 99% of articles on this subject are related to mono- and poly-nuclear complexes of the three mentioned metal ions, with Dy^III^ being the most popular,^12,20−22^ probably because the most promising results are usually presented by dysprosium compounds. Examples are the highest blocking temperature (*T*_B_ = 80 K) reported for the mononuclear Dy^III^ compound [(Cp^i^Pr_5_)Dy(Cp*)][B(C_6_F_5_)_4_], where Cp^i^Pr_5_ = penta-iso-propylcyclopentadienyl and Cp* = pentamethylcyclopentadienyl,^23^ and the larger coercive magnetic field, 14 T, observed for [(Cp^i^Pr_5_)_2_Dy_2_I_3_] at temperatures as high as 60 K.^24^

However, the less studied lanthanide ions, the heavy ones such as Ho^III^, Tm^III^, and Yb^III^ and the lighter Ce^III^ and Nd^III^ ions, can also form lanthanide-based SMMs and exhibit different applications, as highlighted by the recently published reviews by Pointillart et al.^25^ and Borah and Murugavel.^26^ Among them, Ce is the most abundant and inexpensive rare earth element, its natural isotopes do not possess a nuclear spin, and it has been used as a dopant instead of Dy^III^ to improve the magnetic properties of one of the strongest existing permanent magnets, Nd_2_Fe_14_B.^27^ Although the Ce^III^ ion has only one unpaired electron, 4f^1^, magnetic relaxation can also be observed because spin–orbit coupling can create non-negligible magneto-anisotropy. The expected ground state for the oblate Ce^III^ (*L* = 3 and *S* = 1/2) is ^2^F_5/2_, which contributes to its significant magnetic anisotropy. To the best of our knowledge, a total of 11 mononuclear Ce^III^-based SMMs were found, six of them monometallic and five where the Ce^III^ is accompanied by other diamagnetic d transition metal ions (d-Ce^III^). These compounds are collected in Table 1.

**Table 1:** Mononuclear Ce^III^-Based SMMs, Including Monometallic Ce^III^- and d-Ce^III^-Based SMMs. Parameters from the fit of the relaxation time (one line for each different proposed fit) are shown together with the calculated energy difference between ground and first excited Kramer doublets (ΔE) and g_i_ values.

		Orbach		QTM	Raman		direct	calculated		
compound^a^	*H*	*U*_eff_	τ_0_	τ_QTM_	*C*	*n*	*D*	Δ*E*	*g*_*x*_, *g*_*y*_, *g*_*z*_	ref
	/Oe	/cm^–1^	/s	/s	/s^–1^K^–*n*^		/s^–1^K^–1^	/cm^–1^		
Li(DME)_3_[Ce(COT″)_2_]	400	20.85	1.2 × 10^–6^					503	2.43, 2.43, 1.03	(28, 38)
		20.50	1.14 × 10^–6^	0.058						
[Ce(NO_3_)_3_(18-crown-6)]	1000	21.82	1.7 × 10^–7^							(29)
		21.06	2.2 × 10^–7^		0.108	5^b^				
		17.79	9 × 10^–7^		1.5 × 10^–3^	9^b^				
[Ce(NO_3_)_3_(1,10-diaza-18-crown-6)]	1000	30.58	2.3 × 10^–8^							(29)
		31.28	2.6 × 10^–8^		0.52	5^b^				
		15.99	6 × 10^–6^		22	9^b^				
[Ce(NO_3_)_3_(HL_3_)]	3000	26.06	2.76 × 10^–8^	0.076	0.154	7.36		348	0.06, 0.69, 3.82	(30)
[Ce(fdh)_3_(bpy)]	2000	23.14	1.8 × 10^–7^					340	0.18, 0.46, 3.79	(31)
					0.4	6				
[Ce(NO_3_)_3_L^1^_3_]	200	14.94	2.7 × 10^–7^					220	1.90, 1.67, 0.26	(32)
					1.44	6.8	99.5			
[Ce_0.29_La_0.71_(NO_3_)_3_L^1^_3_]	30				0.8	7.55	306.5			
[Ce{Zn(L^2^)}_2_(MeOH)]BPh_4_	0	14.73	1.6 × 10^–7^	0.00038				179.5	0.33, 0.48, 4.06	(33, 38)
[Ce{Zn(L^3^)(AcO)}_2_]BPh_4_	250	25.8	2.7 × 10^–7^							(34)
[Ce(NO_3_){Zn(L^3^) (SCN)}_2_]	1000	24.8	2.2 × 10^–7^							(35)
[CeCd_3_(Hquinha)_3_(*n*-Bu_3_PO)_2_I_3_]	1500			2.58^c^	0.13	6.75	0.0209^c^	303	0.02, 0.10, 2.48	(36)
[Ce(Fcterpy)(NO_3_)_3_(H_2_O)]	2000	13.9	1.8 × 10–11							(37)

a Ligand abbreviations: COT″ = bis(trimethylsilyl)cyclooctatetraenyl dianion, 18-crown-6 = 1,4,7,10,13,16-hexanoxacyclooctadecane, 1,10-diaza-18-crown-6 = 1,4,10,13-tetraoxa-7,16-diazacyclo-octadecane, HL = 2-methoxy-6-[(*E*)-phenylimino-methyl]phenol, fdh = 1,1,1-fluoro-5,5-dimethyl-hexa-2,4-dione, L^1^ = tBuPO(NHiPr)2 = *tert*-butyl-phosphonic-di(isopropylamide), L^2^ = 6,6′-(2,2-dimethylpropane-1,3-diyl)bis(azan-1-yl-1-ylidene)bis(methan-1-yl-1-ylidene) bis(2-meth- oxyphenol), L^3^ = 6,6′-(ethane-1,2-diylbis(azanylylidene)) bis(methanylylidene)bis(2-methoxyphenol). H_2_quinha = quinaldic hydroxamic acid, and Fcterpy = 4′-ferrocenyl-2,2′:6′,2″-terpyridine.

b Fixed value in the fit.

c For comparison purposes, τ_QTM_ and *D* have been calculated by applying the corresponding equation to the values obtained from the fit of the dependence with the field (*B*_1_ = 11.29 s^–1^, *B*_2_ = 1.50 × 10^–6^ Oe^–2^, and *A* = 4.13 × 10^–15^ s^–1^ Oe^–4^ K^–1^).

The monometallic ones include the sandwich compound Li(DME)_3_[Ce(COT″)_2_], where COT″ = bis(trimethylsilyl)cyclooctatetraenyl dianion, reported by Murugesu and coworkers,^28^ and the two air stable compounds, [Ce(NO_3_)_3_(18-crown-6)] and [Ce(NO_3_)_3_(1,10-diaza-18-crown-6)], described by Kajiwara and coworkers, where the 18-crown-6 derivate ligands are equatorially coordinated and the three NO_3_^–^ anions are coordinated to the Ce^III^ in the axial positions.^29^ In addition to the previously mentioned compounds, the following monometallic compounds were also reported: the Ce^III^ Schiff base compound, [Ce(NO_3_)_3_(HL)_3_] {HL = 2-methoxy-6-[(*E*)-phenylimino-methyl]phenol}, reported by the Shanmugam group,^30^ the octacoordinated [Ce(fdh)_3_(bpy)] complex, (fdh = 1,1,1-fluoro-5,5-dimethyl-hexa-2,4-dione), described by Gao and coworkers,^31^ and the compound, [Ce(NO_3_)_3_L_3_] [L = ^*t*^BuP(O)(NH^i^Pr)_2_] with the three P=O donor ligands and the nitrate anions coordinated to the central Ce^III^ ion, described by Murugavel and coworkers, which was also magnetically diluted.^32^

The d-Ce^III^ compounds include the trinuclear compounds [Ce{Zn(L^2^)}_2_(MeOH)]BPh_4_ and [Ce{Zn(L^3^)(AcO)}_2_]BPh_4_ with similar Schiff-base ligands, reported by Kajiwara and coworkers,^33,34^ which are the only SMMs of Ce^III^ revealing the phenomenon without the application on an external direct current (dc) field, although only for the first one, the dynamic susceptibility data could be analyzed at a zero dc field.^33^ Kajiwara and coworkers reported 2 years later another trinuclear CeZn_2_ compound with one of the same Schiff-base, [Ce(NO_3_){Zn(L^3^) (SCN)}_2_], but without showing an SMM behavior in the absence of an external dc field.^35^ Tong and coworkers also reported a nearly perfect hexagonal bipyramidal CeO_8_ geometry in a CeCd_3_ compound, [CeCd_3_(Hquinha)_3_(*n*-Bu_3_PO)_2_I_3_], which was achieved by the assembly of a 15-MC-6 (metallocrown) and the axial coordination of phosphine oxides.^36^ Lastly, Wang and coworkers reported the [Ce(Fcterpy)(NO_3_)_3_(H_2_O)] compound, where they employed a ferrocenyl terpyridine derivate.^37^

To evaluate the spin relaxation in SMMs, several mechanisms should be considered, such as the Orbach mechanism, the one-phonon direct process, the two-phonon Raman process, the local-mode process, and the quantum tunneling of magnetization (QTM).^39,40^ In the previously reported mononuclear Ce^III^ SMM compounds, several approaches have been employed, see Table 1 and references herein. Usually, the initial evaluation of the relaxation time considering only an Orbach process is performed. For most of the complexes in Table 1, an external dc field is needed to present slow relaxation of the magnetization, and when evaluating the spin relaxation in some cases, only the “optimal external field is determined”, whereas in other occasions, the dependence of the relaxation time (τ) with the applied external field is discussed in more detail. However, the dependence of τ with the external field has only been fit in one case.^36^ On the other hand, the relaxation mechanisms usually employed to fit the dependence of τ with temperature at a specific external dc field are Orbach, QTM, one-phonon direct, and two-phonon Raman processes. Moreover, it is worth noting that in any of the cases, the calculated anisotropic barrier (ΔE in Table 1) coincides with the obtained energy barrier in the different fits. In general, the calculated energy barrier is 1 order of magnitude bigger, which is an indication that an Orbach process, which should involve a real state, is not the primarily responsible process for the spin relaxation as the energy of the first excited state is not close to the energy barrier obtained from the fit. Therefore, a deeper evaluation of the spin relaxation is needed.

In the synthesis of the Ce^III^ complexes shown above, different ligands have been employed. The β-diketone ligands in combination with polypyridine derivatives have been strongly emerged into this area.^41−43^ Besides the variety, typically two categories of complexes were produced when Ln^III^ ions are coordinated with the anionic β-diketone ligands, the neutral complexes in the 1:3 stoichiometric ratio or the anionic complexes in the 1:4 ratio.^41^ In the field of molecular magnetism, since the discovery of mononuclear [Dy(acac)_3_(H_2_O)_2_] (acac = acetylacetonate) behaving as an SMM,^42^ most of the research of Ln^III^/β-diketonate has been focused on Dy^III^ ions. In the case of octa-coordinated complexes, a square antiprism (D_4d_) symmetry was most likely constructed, a geometry that can be beneficial for the maximization of magnetic anisotropy with the appropriate ligand field. The same symmetry was also generated when the Ln^III^ ions and β-diketonate with different substituents are combined with auxiliary N-donors polypyridyl ligands. This strategy was employed to yield mononuclear Dy^III^ SMMs with a D_4d_ symmetry with the aim of obtaining magnetostructural correlations regarding electronic effects and intermolecular interactions.^43−47^ This approach was successfully used by some of us to synthesize several pyridyl adducts, [Ln^III^(β-diketonate)_3_(NN)], where NN = polypyridyl ligands,^48−50^ in which the corresponding mononuclear pyridyl adducts of Nd^III^ showed an SMM behavior.^50^

**Scheme 1:**
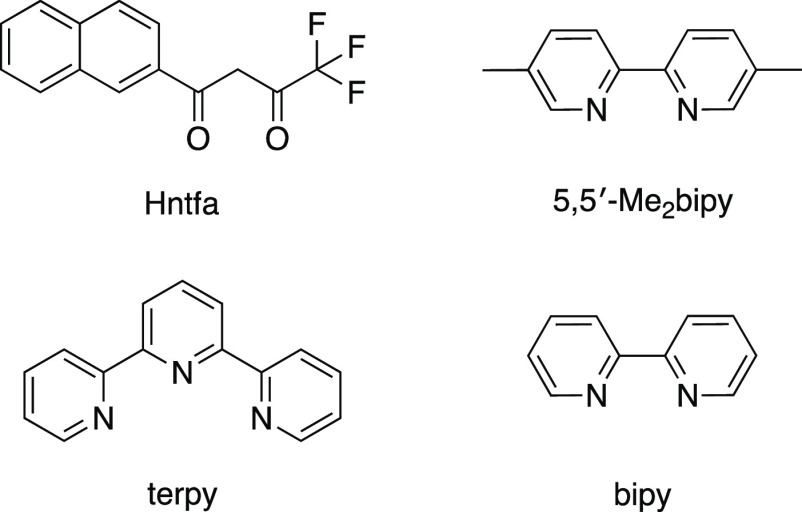
Structural Formulas of Ligands Used in This Study

Regarding Ce^III^ complexes displaying an SMM behavior (Table 1), the only pyridyl adduct with a β-diketonate ligand was the mononuclear octacoordinated [Ce(fdh)_3_(bpy)] compound.^31^ Therefore, this study was undertaken to synthesize some polypyridyl adducts of Ce^III^–Hntfa complexes and investigate their magnetic properties and SMM behavior. Four mononuclear Ce^III^ compounds with the formulas [Ce(ntfa)_3_(MeOH)_2_] (**1**), [Ce(ntfa)_3_(5,5′-Me_2_bipy)] (**2**), [Ce(ntfa)_3_(terpy)] (**3**), and [Ce(ntfa)_3_(bipy)_2_] (**4**) have been structurally derived from Hntfa = 4,4,4-trifluoro-1-(naphthalen-2-yl)butane-1,3-dione and some polypyridyl auxiliary ligands, including 5,5′-Me_2_bipy = 5,5′-Dimethyl-2,2′-dipyridine, terpy = 2,2′:6′,2″-terpyridine, and bipy = 2,2′-bipyridine, and characterized (Scheme 1).

## Experimental Section

### Materials and General Procedures

4,4,4-Trifluoro-1-(2-naphthyl)-1,3-butanedione, 2,2′-bipyridine, 2,2′:6′,2″-terpyridine, and 5,5′-dimethyl-2,2′-dipyridine were purchased from TCI, cerium(III) nitrate hexahydrate was purchased from Sigma Aldrich, and other chemicals were of analytical grade quality. Attenuated total reflection infrared (ATR-IR) spectra of solid complexes were recorded on a Bruker Alpha P spectrometer. C, H, and N elemental microanalyses were performed using an Elementar Vario EN3 analyzer. Phase purity of the solid bulk material of the title compounds was checked using a Bruker D8 ADVANCE X-ray powder diffractometer (CuKα radiation).

### Synthesis and Characterization

#### [Ce(ntfa)_3_(MeOH)_2_] (**1**)

A methanolic solution (10 mL) of Ce(NO_3_)_3_·6H_2_O (261 mg, 0.60 mmol) was added to a mixture containing 4,4,4-trifluoro-1-(2-naphthyl)-1,3-butanedione (495 mg, 1.86 mmol) and 1 M NaOH (1,8 mL) in MeOH (20 mL). After 2 h of stirring the reaction mixture, 40 mL of deionized water was added, and the mixture was stirred for 12 h at ambient temperature and then filtered off. The obtained orange powder was dried at 80 °C for 30 min (yield: 473 mg, 81%). Anal. Calcd. for C_44_H_32_CeF_9_O_8_: C, 52.9; H, 3.2. Found: C, 52.8; H, 3.2%. Selected IR bands (ATR-IR, cm^–1^): 2916 (s), 1607 (s), 1567 (m), 1531 (m), 1508 (m), 1454 (m), 1425 (w), 1386 (w), 1351 (w), 1285 (s), 1251 (m), 1220 (w), 1186 (s), 1126 (s), 1071 (m), 956 (m), 864 (m), 793 (s), 718 (m), 728 (m), 682 (s), 567 (m), 518 (w), 469 (m), 452 (w), 396 (w).

#### [Ce(ntfa)_3_(5,5′-Me_2_bipy)] (**2**)

[Ce(ntfa)_3_(MeOH)_2_] (120 mg, 0.12 mmol) was dissolved in 15 mL of ethanol. 5,5′-Dimethyl-2,2′-dipyridyl (57 mg, 0.31 mmol) was dissolved in ethanol (15 mL). The solutions were combined and stirred for approximately for 2 h. The mixture was filtered and allowed to be at room temperature. After 2 days, the resulting red crystals were collected by filtration and allowed to dry in air (yield: 74 mg, 46%). Anal. Calcd. for C_54_H_36_CeF_9_N_2_O_6_: C, 57.9; H, 3.2; N, 2.5. Found: C, 58.0; H, 3.1; N 2.4%. Selected IR bands (ATR-IR, cm^–1^): 1608 (s), 1589 (m), 1567 (m), 1528 (m), 1506 (w), 1460 (m), 1383 (w), 1354 (w), 1282 (s), 1217 (w), 1197 (m), 1126 (s), 1071 (m), 955 (m), 921 (w), 867 (w), 792 (s), 752 (m), 680 (m), 565 (m), 518 (w), 484 (m), 391 (w).

#### [Ce(ntfa)_3_(terpy)] (**3**)

[Ce(ntfa)_3_(MeOH)_2_] (151 mg, 0.15 mmol) was dissolved in 15 mL of ethanol/acetone (3:1), and 2,2′:6′,2″-terpyridine (39 mg, 0.17 mmol) was dissolved in 15 mL of ethanol/acetone (3:1). The two solutions were combined and stirred for approximately for 3 h at 70 °C. The mixture was filtered off and allow to crystallize at room temperature. After 2 days, the orange crystals, which were separated, were collected by filtration and dried in air (yield: 66 mg, 37.5%). Anal. Calcd. for C_57_H_35_CeF_9_N_3_O_6_: C, 58.6; H, 3.0; N, 3.6. Found: C, 58.3; H, 3.1; N 3.6%. Selected IR bands (ATR-IR, cm^–1^): 1611 (s), 1593 (m), 1569 (m), 1526 (m), 1506 (m), 1476 (m), 1386 (w), 1352 (w), 1285 (s), 1217 (w), 1183 (m), 1122 (s), 1069 (m), 1009 (m), 955 (m), 936 (w), 865 (m), 789 (s), 763 (m), 681 (m), 564 (m), 518 (w), 476 (m), 391 (w).

#### [Ce(ntfa)_3_(bipy)_2_] (**4**)

[Ce(ntfa)_3_(MeOH)_2_] (270 mg, 0.27 mmol) was dissolved in 30 mL of ethanol. 2,2′-Bipyridine (98 mg, 0.63 mmol) was dissolved in 10 mL of ethanol. The solutions were combined, stirred for approximately 2 h, filtered, and then allowed to crystallize at room temperature. After 2 days, the red crystals were separated, collected by filtration, and dried in air (yield: 132 mg, 46%). Anal. Calcd. for C_62_H_40_CeF_9_N_4_O_6_: C, 59.7; H, 3.2; N, 4.5. Found: C, 59.6; H, 3.3; N 4.4%. Selected IR bands (ATR-IR, cm^–1^): 1632 (m), 1614 (s), 1592 (m), 1567 (m), 1504 (m), 1467 (m), 1433 (w), 1385 (w), 1352 (w), 1283 (s), 1166 (m), 1004 (m), 958 (m), 925 (w), 862 (w), 816 (w), 785 (s), 751 (s), 680 (s), 636 (w), 563 (m), 505 (w), 471 (m), 447 (w), 414 (m).

### Single-Crystal Structural Determination

Suitable single crystals of complexes **1–4** were selected by use of a polarizing microscope and mounted on an Bruker-AXS APEX CCD diffractometer. Data collection of X-ray single-crystal data was performed at 100(2) K by use of Mo-Kα radiation (λ = 0.71073 Å), and the data were subsequently processed (Lp, and absorption corrections; APEX and SADABS).^51^ The structures were solved and refined using the SHELX package (direct methods; full-matrix least-squares on F^2^).^52^ Anisotropic displacement parameters were applied for all non-hydrogen atoms. The hydrogen atoms were located from difference Fourier maps, assigned with isotropic displacement factors, and included in the final refinement cycles by use of geometrical constraints. Other programs, Mercury,^53^ Olex,^54^ and Platon,^55^ were also employed. Main parameters, data, and refinement details are collected in Table 2.

**Table 2:** Crystal Data and Structural Refinement of **1–4**

compound	**1**	**2**	**3**	**4**
empirical formula	C_44_H_32_CeF_9_O_8_	C_54_H_36_CeF_9_N_2_O_6_	C_57_H_35_CeF_9_N_3_O_6_·solvent	C_62_H_40_CeF_9_N_4_O_6_
formula weight	999.82	1119.97	1169.00	1248.10
crystal system	monoclinic	orthorhombic	monoclinic	triclinic
space group	*P*2_1_/*c*	*Pca*2_1_	*C*2/*c*	*P*1̅
temp (K)	100(2)	100(2)	100(2)	100(2)
*a* (Å)	8.9526(9)	19.7890(8)	43.911(2)	11.9134(8)
*b* (Å)	28.977(2)	13.5105(6)	11.1912(5)	15.3600(8)
*c* (Å)	16.2989(16)	18.1551(6)	41.9824(17)	16.5659(10)
α (deg)	90	90	90	79.120(3)
β (deg)	105.509(5)	90	92.594(4)	70.343(4)
γ (deg)	90	90	90	67.664(3)
*V*/Å^3^	4074.3(6)	4853.9(3)	20609.7(16)	2634.1(3)
*Z*	4	4	16	2
*D* (calcd)(g/cm^3^)	1.630	1.533	1.507	1.574
μ (mm^–1^)	1.212	1.025	0.970	0.955
*F*(000)	1996.0	2244.0	9360.0	1254.0
λ (Å)	0.71073	0.71073	0.71073	0.71073
GOF on *F*^2^	1.027	1.042	1.055	1.091
*R* (*I* > 2σ(*I*))	0.0482	0.0252	0.0612	0.0529
w*R*_2_ (all data)	0.0874	0.0638	0.1603	0.1130

### Magnetic Measurements

A Quantum Design MPMS XL SQUID magnetometer was employed. Data were collected for powder microcrystalline samples or for crushed polycrystalline samples of complexes **1–4** in a gelatin capsule, whose purity and structural integrity were analyzed by powder X-ray diffraction (Figures S1–S4). dc susceptibility measurements were acquired between 2 and 300 K and under an external magnetic field of 0.3 T. For the capsule and holder, blank measurements were performed, and their diamagnetic contributions were corrected. Diamagnetic corrections of the complexes were estimated from Pascal tables.^56^ Alternated current (ac) susceptibility measurements were carried out by applying an oscillating ac field of 4 Oe with ac frequencies between 1 and 1500 Hz at different temperatures and dc applied fields indicated in the text.

### Computational Details

Ab initio calculations were performed using the OpenMolcas package,^57^ version 18.09, and the crystallographic geometries. The MOLCAS ANO-RCC basis set^58−60^ was employed for all atoms with the following contractions: Ce [9s8p6d4f3g2h], O[4s3p2d1f], N[4s3p2d1f], F[3s2p], C[3s2p], and H[2s]. The CASSCF method was used to calculate the spin-free state energies of the compounds, and the spin–orbit coupling was included perturbatively in the second step by using the restricted active space state interaction (RASSI) method.^61^ Due to the large ionic character of the Ln–O and Ln–N bonds, dynamic correlation contributions are not essential, which was also demonstrated for Ce complexes,^38^ and CASPT2 calculations were not performed. In the CASSCF calculation, a (1,7) active space was used considering the seven doublets. The SINGLE_ANISO module, as implemented in OpenMolcas, was employed for the evaluation of the direction and magnitude of the magnetic moments of the final states.^62,63^ Also, the probability of transition between different states has been estimated by the calculation of the matrix elements of the transition magnetic moments. Additionally, experimental geometries were optimized, and vibrational frequencies were calculated using the Gaussian09 D01 code^64^ with the B3LYP functional^65^ using the Stuttgart pseudo/basis set^66^ for Ce and a TZV basis set^67^ for lighter atoms.

## Results and Discussion

### Synthesis

The interaction of a methanolic solution containing Ce(NO_3_)_3_·6H_2_O, 4,4,4-trifluoro-1-(2-naphthyl)-1,3-butanedione (Hntfa), and NaOH in the stoichiometric ratio of 1:3:3 afforded the neutral complex [Ce(ntfa)_3_(MeOH)_2_] (**1**). This complex was used as a precursor for the synthesis of polypyridyl adducts **2-4**. The reaction of **1** with 5,5′-Me_2_bipy in ethanol and terpy in the ethanol/acetone (3:1) mixture resulted in the formation of the mononuclear complexes [Ce(ntfa)_3_(5,5′-Me_2_bipy)] (**2**) and [Ce(ntfa)_3_(terpy)] (**3**), respectively, through the substitution of the two coordinated MeOH molecules in **1** by one molecule of the corresponding polypyridyl derivatives and resulting in complexes with coordination numbers (CNs) of 8 and 9, respectively. Unlike the mono-pyridyl adducts **2** and **3**, the reaction of **1** with bipy in ethanol with a 1:2 stoichiometric ratio afforded the bis-bipy product [Ce(ntfa)_3_(bipy)_2_] (**4**) with a CN of 10 (see the next section). Attempts with 1:1 stoichiometry afforded the same bis-bipy product **4**. The procedure described here for the synthesis of the pyridyl adducts was successfully demonstrated with other lanthanide ions (Ln^III^ = La^III^, Pr^III^, Ho^III^, and Nd^III^) and β-diketone derivatives.^48−50^ The four complexes were characterized by elemental microanalyses and IR spectroscopy, and their molecular structures were determined by single-crystal X-ray crystallography. In addition, the purity of the microcrystalline solids was characterized by PXRD (Figures S1–S4, Supporting Information). The IR spectra of the complexes revealed their characteristic coordination feature, where the strong vibrational band observed at around 1610 cm^–1^ for **1–3** complexes and at 1632 cm^–1^ in the case of **4** is typically assigned to the stretching frequency, ν(C=O), of the coordinated carbonyl group of ntfa.

### Crystal Structures

**Figure 1:**
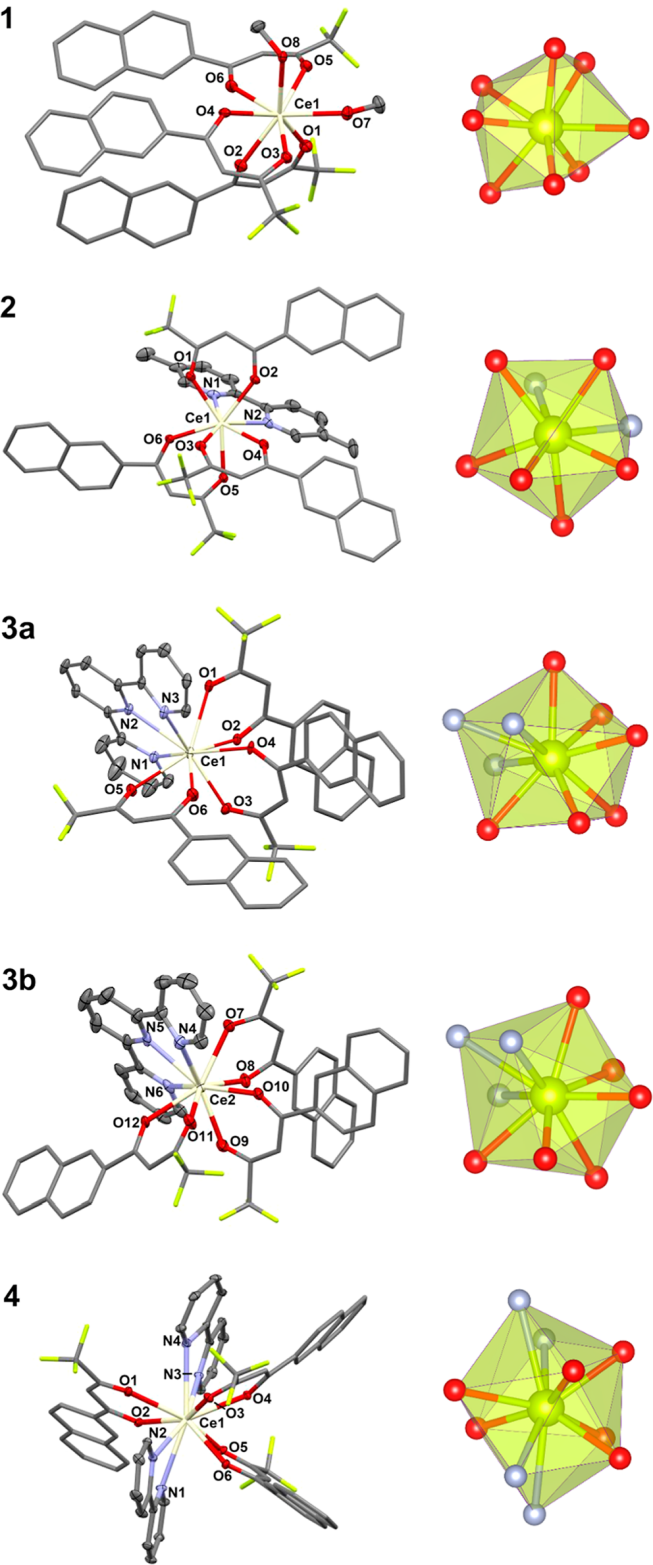
Perspective view (left) and coordination figure (right) of compounds **1**–**4**. Selected bond distances and angles are collected in Table 3.

Each Ce^III^ center in the monomeric neutral complexes **1–4** is ligated by six oxygen donor atoms of three β-diketonato ligand anions (ntfa) (Figure 1). A CN of 8 around Ce1 in **1** is completed by oxygen atoms of two terminal methanol molecules and in **2** by two N-donor atoms of the chelating 5,5′-dimethylbipyridine molecule. Selected distances and angles are collected in Table 3. The Ce–N/O bond distances in **1** and **2** are in the range from 2.398(2) to 2.671(3) Å. The CeO_6_N_3_ “chromophore” around the two crystallographic independent Ce^III^ centers in **3** is formed by ligation of one terpy chelating ligand, achieving a CN of 9 around the central Ce^III^ ion. The two crystallographic independent molecules in **3** differ mainly in the opposite “head-to-tail” arrangement of one diketo anion (i.e., that with O5 and O6 bonded to Ce1 compared to that with O11 and O12 bonded to Ce2). The Ce–N/O bond distances in **3** are in the range from 2.431(6) to 2.703(6) Å. A CN of 10 around Ce1 in **4** is formed by four nitrogen donor atoms of two bipy ligands with Ce–N/O bond distances in the range from 2.465(3) to 2.847(4) Å. The O–Ce–O bite angles of the β-diketonato groups fall in the range from 67.16(10) to 71.02(7)° in **1–4**, respectively, and the N–Ce–N bite angles of the chelating 5,5′-Me_2_bipy, terpy, and bipy molecules in **2–4** complexes vary from 56.80(10) to 61.1(2)°.

**Table 3:** Selected Bond Distances (Å) and Angles (°)

compound	**1**	**2**	**3-Ce1**	**4**
/fragment			[**3-Ce2**]	
Ce1–O1	2.427(2)	2.456(2)	2.476(4)	2.530(3)
[Ce2–O7]			[2.511(5)]	
Ce1–O2	2.398(2)	2.415(2)	2.538(4)	2.478(3)
[Ce2–O8]			[2.484(5)]	
Ce1–O3	2.493(2)	2.419(2)	2.467(4)	2.494(3)
[Ce2–O9]			[2.431(6)]	
Ce1–O4	2.465(2)	2.455(2)	2.457(4)	2.465(3)
[Ce2–O10]			[2.440(5)]	
Ce1–O5	2.421(2)	2.432(2)	2.470(5)	2.474(3)
[Ce2–O11]			[2.488(5)]	
Ce1–O6	2.438(2)	2.451(2)	2.497(5)	2.475(3)
[Ce2–O12]			[2.454(4)]	
Ce1–O7/Ce1–N1	2.518(3)	2.671(3)	2.621(5)	2.795(4)
[Ce2–N4]			[2.630(6)]	
Ce1–O8/Ce1–N2	2.609(2)	2.671(3)	2.686(5)	2.756(4)
[Ce2–N5]			[2.703(6)]	
Ce1–N3			2.645(5)	2.811(3)
[Ce2–N6]			[2.680(6)]	
Ce1–N4				2.847(4)
O1–Ce1–O2	69.28(8)	68.66(8)	68.62(14)	67.16(10)
[O7–Ce2–O8]			[72.20(18)]	
O3–Ce1–O4	68.35(8)	69.52(8)	73.70(14)	68.20(9)
[O9–Ce2–O10]			[66.86(16)]	
O5–Ce1–O6	70.14(8)	71.02(7)	67.88(15)	68.75(10)
[O11–Ce2–O12]			[67.87(16)]	
N1–Ce1–N2		60.63(9)	60.87(16)	57.90(11)
[N4–Ce2–N5]			[61.1(2)]	
N3–Ce1–N2			61.59(15)	
[N5–Ce2–N6]			[60.2(2)]	
N3–Ce1–N4				56.80(10)

The SHAPE software^68,69^ was used to determine the degree of distortion of the coordination polyhedra in complexes **1–4**. Intermediate distortion is observed between various ideal eight-vertex coordination polyhedra for the CeO_8_ and CeO_6_N_2_ coordination polyhedra of **1** and **2**, between various ideal nine-vertex coordination polyhedra for the CeO_6_N_3_ coordination polyhedron of **3**, and between various ideal 10-vertex coordination polyhedra for the CeO_6_N_4_ coordination polyhedron of **4**, respectively. The lowest continuous shape measurement (CShM) values for **2** correspond to the triangular dodecahedron (TDD-8), square antiprism (SAPR-8), biaugmented trigonal prism (BTPR-8), and biaugmented trigonal prism J50 (JBTPR-8) with values of 1.269, 1.407, 2.420, and 2.939; whereas for **1**, the following values were found: 0.412, 2.532, 1.732, and 2.782, respectively. The lowest CShM values for compound **3** correspond to the spherical capped square antiprism (CSAPR-9), muffin (MFF-9), spherical tricapped trigonal prism (TCTPR-9), and capped square antiprism J10 (JCSAPR-9) with values of 0.973, 1.278, 2.074, and 2.119 for Ce1 and 1.786, 1.263, 2.472, and 2.866 for Ce2, respectively. For **4**, the lowest CShM value corresponds to sphenocorona J87 (JSPC-10) with a value of 0.478. The next lowest value of 4.083 corresponds to the bicapped square antiprism J17 (JBCSAPR-10).

In **1**, centrosymmetric dimers with a Ce···Ce separation of 5.536 Å are formed via O–H···O hydrogen bonds [O7–H10···O8′ = 162(3)°; O7···O8′ = 2.748(3) Å; O8–H20···O3′ = 160(3)°, and O8···O3 = 2.693(3) Å; (′) = 1 – *x*,1 – *y*,1 – *z*)]; whereas in **2–4**, no classical hydrogen bonds exist. The shortest Ce···Ce distances observed in **2–4** are 10.996, 13.385, and 9.963 Å, respectively.

Aside from the observed different coordination, the coligands also favor or avoid different intermolecular interactions. The pyridyl rings form interplanar angles with their mean planes of 14.9 and 5.6° for the two bipy molecules in **4** and 6.2° in the case of the 5,5′-Me_2_bipy molecules in **2**. In **3**, the pyridyl rings of N1(N5) and of N2(N6) are practically co-planar with an interplanar angle of 2.4(4.0)°, whereas the pyridyl ring of N3(N4) forms an interplanar angle of 22.0(29.0)° with the central pyridyl ring of the terpy ligand. The pyridyl- (in **2–4**) and naphthyl- (in **1–4**) aromatic ring systems are involved in C–H/F···ring and ring···ring interactions (Supporting Information, Tables S1–S4). Among them, the short ring···ring interactions, with the separation of their centers of gravity (Cg) being less than 3.83 Å, observed are as follows: in **1**, between naphthyl rings (C19–C28) and (C33–C42) [*x*,1/2 – *y*,1/2 – *z*]; in **2**, between the pyridyl ring (N2,C49–C53) and naphthyl ring (C5–C14) [1/2 – *x*,*y*,–1/2 + *z*]; in **3**, between pyridyl rings (N5,C105–C109) and (N6,C110–C114) [1 – *x*,1 – *y*,1 – *z*], between the pyridyl ring (N3, C57–C63) and naphthyl ring (C19–C28) [*x*,1 + *y*,*z*], between naphthyl rings (C61–C69,C122) and (C33–C42), and between naphthyl rings (C74–C83) and (C74–C83) [1 – *x*,*y*,1/2 – *z*]; in **4**, between pyridyl rings (N3,C53–C57) and (N3,C53–C57) [1 – *x*,1 – *y*,1 – *z*] and between naphthyl rings (C33–C42) and (C33–C42)[−*x*,–*y*,2 – z].

### Magnetic Characterization

**Figure 2:**
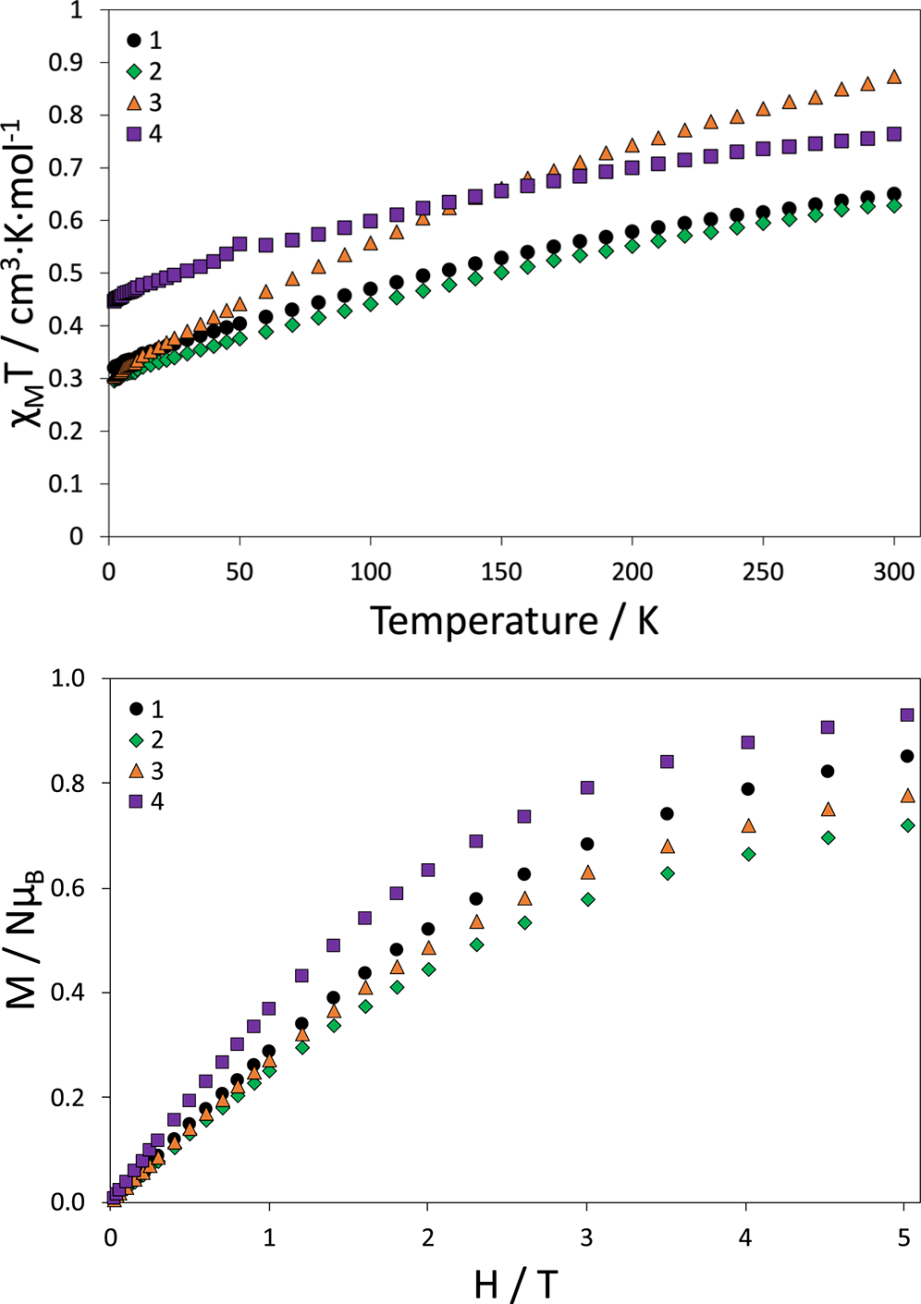
(top) χ_M_*T* vs *T* plot and (bottom) *M* vs *H* plot for compounds **1–4**.

dc magnetic susceptibility and magnetization measurements were performed for compounds **1–4** on polycrystalline samples. The susceptibility curves in the 2–300 K temperature range are shown in Figure 2 (top). At room temperature (300 K), the χ_M_*T* values for compounds **1–4** are 0.650, 0.628, 0.874, and 0.764 cm^3^·mol^–1^·K, respectively. These values are close to the ones expected for one isolated Ce^3+^ cation at the ground state (^2^F_5/2_), which is 0.8 cm^3^·mol^–1^·K. On cooling the samples, the χ_M_*T* values decrease gradually to the values of 0.333, 0.309, 0.318, and 0.465 cm^3^·mol^–1^·K, respectively, at 2 K, which can be ascribed to the progressive depopulation of the *m*_J_ states. The field dependence of magnetization (*M*) of these compounds was recorded at 2 K, Figure 2 (bottom). On increasing the external magnetic field up to 5 T, the M values increase to 0.850, 0.721, 0.776, and 0.929 *N*μ_B_ for compounds **1–4**, respectively. Despite the gradual increase at a high field, no saturation of the magnetization is observed, which indicates significant anisotropy.

**Figure 3:**
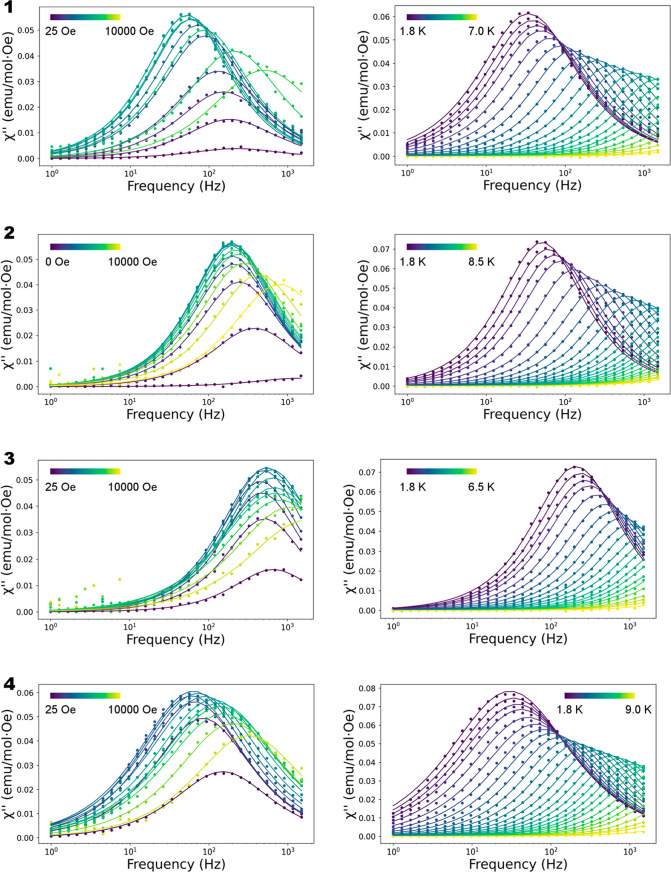
Out-of-phase (χ_M_″) component of the frequency dependence ac susceptibility for compounds **1–4**. (Left) Field dependence acquired at 2.5 K and (right) temperature dependence acquired at 200 Oe. Solid lines are the result of the fit of the data to a Debye function using the CCfit package.

In order to study the dynamic magnetic properties and the possible SMM behavior, ac magnetic susceptibility measurements with variable values of the field, temperature, and frequency were recorded for compounds **1–4** under the same conditions, as depicted in Figure 3. Without the application of an external dc field, none of these compounds exhibited dependence of the out-of-phase (χ_M_″) signal with frequency and temperature, although a small signal seems to start for **2**. This fact suggests that the relaxation of the magnetization under these conditions occurs through fast QTM. The application of a dc magnetic field of only 25 Oe is enough to suppress the QTM for **2–4**, and a clear maximum appears in the χ_M_″ component. In the case of **1**, a slightly larger field is needed, 50 Oe. The need of a larger dc field for **1** might be correlated with the shorter Ce···Ce distance, 5.536 Å (vs 10.996, 13.385, and 9.963 Å for **2–4**, respectively) and the presence of the intramolecular hydrogen bond found in **1**. In all the compounds, upon increasing the applied dc field, the maximum first moves to smaller frequencies, indicating slower relaxation times (τ); then, the maximum position appears in similar frequency values for a range of fields and then finally moves to larger frequencies. Although the general tendencies can be relatively similar, compound **1** covers a large range of frequencies and has the slower τ, followed by **4**, **2**, and **3**.

To also analyze the dependence of χ_M_″ on temperature, the same external dc field was selected for all the complexes. In this case, 200 Oe was selected as a compromise for all the compounds, being the one where in general the maximum of χ_M_″ had a larger value and the relaxation time was larger. The temperature dependence of χ_M_″ with frequency shows the typical SMM behavior. The maximum in the out-of-phase component of the susceptibility appears between 10 and 1000 Hz depending on the compound and moves to larger frequencies when the temperature is increased.

In order to analyze the spin dynamics of these Ce^III^ compounds, the Cole–Cole plots were fitted to a Debye function using the CCfit package (Figures S9–S24), and relaxation times and α parameters have been extracted from the experimental data (Tables S1–S8). The α values are in general small, indicating a relatively narrow distribution of relaxation times. In general, maximum values (0.18, 0.13, 0.24, and 0.26 for **1**, **2**, **3**, and **4**, respectively) are obtained at larger fields or smaller temperatures. The spin–lattice relaxation rate τ parameter can be employed to analyze the spin relaxation mechanism by analyzing the dependence of τ^–1^ with *T* and the applied external field, which follows eq 1
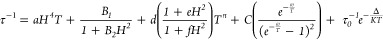
1

The terms in eq 1 refer to direct, QTM, Raman, local-mode, and Orbach relaxation mechanisms, in the given order. The Raman term is a field-dependent term using the Brons–van Vleck equation, it has a coefficient field-dependent (*d* represents zero-field relaxation, *e* is related with paramagnetic center concentration, and the *f* parameter reports the effect of the external field in suppressing the spin relaxation) and also the typical exponential dependence with the temperature. The Orbach processes are not considered because the ab initio calculations (see the next section) show that the first excited state (250–300 cm^–1^) is much higher in energy than the obtained energy barrier using the last term of eq 1 (around 10–20 cm^–1^). In fact, for all the reported systems (Table 1), the energy barrier oscillates between 15 and 32 cm^–1^, while none of the calculated systems have an excited state so close in energy. To analyze the dependence of τ^–1^ with *T* and field, we first focus on the field dependence because despite the presence of several mechanisms, usually one of them (among the three first terms of eq 1, which are the ones having field dependence) is the predominant in each part of the curve.

**Figure 4:**
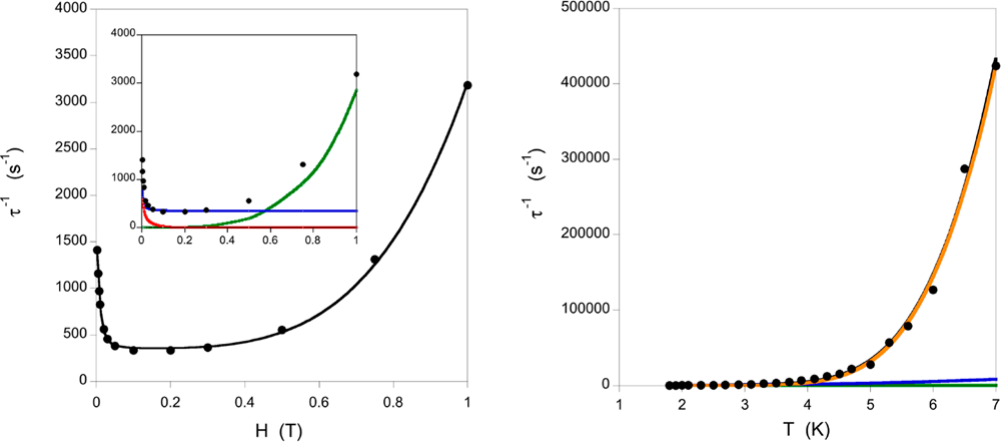
Dependence of the inverse of the spin relaxation time for **1** (left) on a static external field at 2.5 K and (right) on the temperature with a 0.02 T external field. The black solid line represents the fit of the experimental data (black dots) with the terms of eqs 2 and 3, respectively. In the inset of the field dependence, green, blue, and red lines correspond to the direct, Raman, and tunneling contributions, respectively. In the temperature dependence (right), green, blue, red, and orange lines correspond to the direct, Raman, tunneling, and local-mode (superposed with the black line) contributions, respectively.

As represented in Figure 4, at low external fields, the tunneling mechanism is responsible of the fast τ^–1^ decay. For intermediate field values, the field-dependent Raman mechanism is the main contribution, whereas at high field values, the direct mechanism becomes predominant due to the H^4^ dependence (see Figure 4). Hence, each region can be independently fitted with the corresponding term (see eq 2), and these parameters are the starting point for the fitting of the whole set. The obtained final values for **1** are *a* = 2855 s^–1^ T^–4^ K^–1^, *B*_1_ = 606 s^–1^, *B*_2_ = 6312 T^–2^, *dT*^*n*^ = 967 s^–1^, *e* = 16662 T^–2^, and *f* = 46153 T^–2^ (see Figure 4 left).
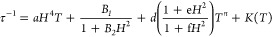
2

The dependence with the temperature can be fitted using eq 3, where the three field-dependent constants are those determined using the fitted parameters from eq 2, and thus, there are only three parameters to be fit (*n*, *C*, and ω). Despite the lower number of parameters, the temperature dependence is more complicated because the last two terms containing these three parameters have a very similar numerical dependence, and both can be predominant at high temperatures. However, for **1**, the field dependence values indicate a relatively small τ^–1^ value in the predominant Raman region (around 0.2–0.4 T in Figure 4 left). This fact results in a very small contribution of this Raman term in the temperature dependence; thus, the temperature dependence is basically due to the local mode (see Figure 4 right). The fit of the temperature dependence curve using eq 3 resulted in the final values of the *n* exponent of the Raman term being *n* = 2.98 and the two parameters of the local-mode relaxation being *C* = 1.23 × 10^9^ s^–1^ and ω = 46.4 K (32.2 cm^–1^).
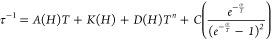
3

The analysis of the density functional theory (DFT) vibrational frequencies for **1** allows us to identify two modes at 24.3 and 25.0 cm^–1^ (close to the fitted local-mode value of 32.2 cm^–1^) as those with largest reduced mass, indicating a large contribution of the lanthanide atom. A spin-phonon calculation corresponding to such vibrational modes would verify their participation in the spin relaxation, but it is out of the scope of the paper.

**Figure 5:**
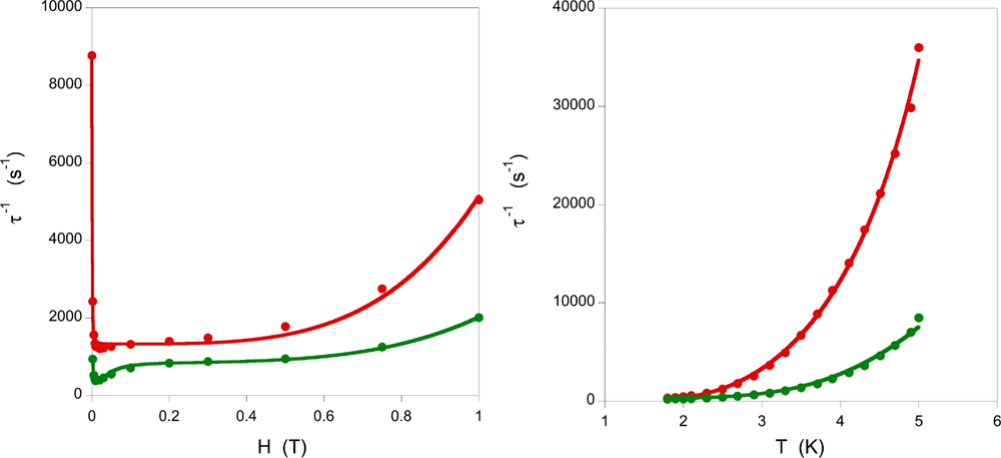
Dependence of the inverse of the spin relaxation time for **2** (red) and **4** (green) on a static external field at 2.5 K and (right) on the temperature with a 0.02 T external field. The solid line represents the fit of the experimental data (dots) with the terms of eqs 2 and 3, respectively.

The same fitting procedure has been performed for the other two compounds containing bipyridine-type ligands (except for **3** because of the presence of two different molecules in the unit cell), and the results are illustrated in Figure 5. The fitted values for **2** are *a* = 3811 s^–1^ T^–4^ K^–1^, *B*_1_ = 6347 s^–1^, *B*_2_ = 9.24 × 10^5^ T^–2^, *dT*^*n*^ = 2426 s^–1^, *e* = 7.34 × 10^5^ T^–2^, and *f* = 1.34 × 10^6^ T^–2^ for the field dependence (eq 2) and *n* = 8.01, *C* = 2.83 × 10^5^ s^–1^, and ω = 13.6 K (9.5 cm^–1^) (eq 3), while for **4**, the values are *a* = 1162 s^–1^ T^–4^ K^–1^, *B*_1_ = 1177 s^–1^, *B*_2_ = 1.02 × 10^5^ T^–2^, *dT*^*n*^ = 209 s^–1^, *e* = 2390 T^–2^, and *f* = 583 T^–2^ for the field dependence (eq 2) and *n* = 1.28, *C* = 6.20 × 10^5^ s^–1^, and ω = 22.7 K (15.7 cm^–1^) (eq 3).

The comparison of the results indicates the following: (i) the importance of tunneling relaxation on these complexes, which can be eliminated with very small external fields. The field needed is smaller for **2** (which starts to show a signal at zero field) and increases for **4** and **1**, being larger the field required for **1** than the required for **4**. This might be related with the Ce···Ce distances being larger in **2** and **4** than in **1** (5.536 Å in **1**, 10.996 in **2**, and 9.963 Å in **4**) and the different intermolecular interactions (hydrogen bond in **1**, one short ring···ring interaction in **2**, and two short ring···ring interactions in **4**) and packing. Thus, the larger dimethyl bipyridine ligands of **2** adopt a less dense packing, resulting in a smaller dipolar interaction and consequently less efficient tunneling relaxation. (ii) τ^–1^ is smaller for **1** and **4** at intermediate fields, indicating a less effective Raman spin relaxation for such systems. (iii) The direct term gives larger τ^–1^ contributions at high fields for **2**, followed by **1**. (iv) The faster temperature-dependent spin relaxation of **2** is basically due to the two main spin relaxation mechanisms, Raman and local-mode, playing an active role, while in **1** and **4**, only the local-mode mechanism is significant, resulting in slower relaxation.

### Computational Results

**Figure 6:**
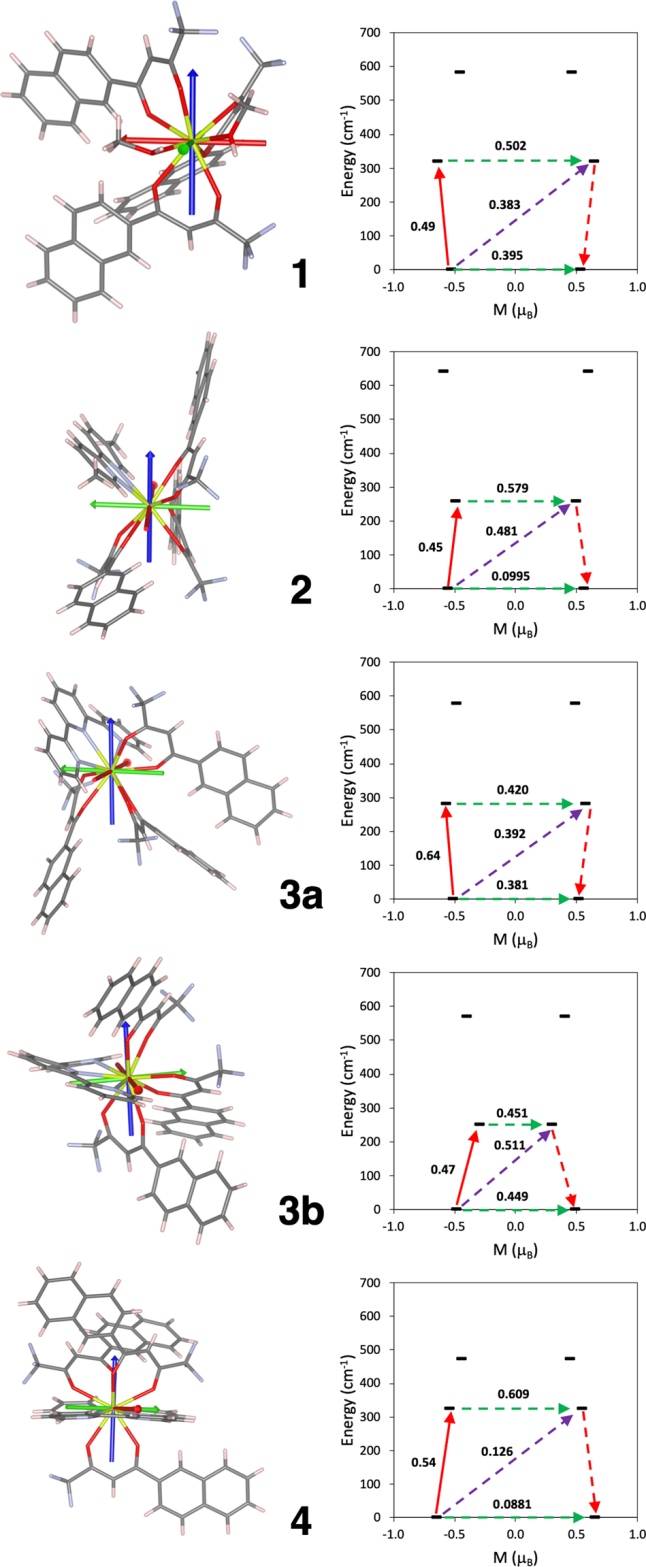
(left) Calculated ab initio orientations of the g tensor of the ground Kramers doublet. Blue, green, and red arrows represent *g*_*z*_, *g*_*y*_, and *g*_*x*_ components, respectively. The atoms abide by the following color code: yellow, cerium; red, oxygen; blue, nitrogen; gray, carbon; and pink, hydrogen. (right) Energies of states as a function of their magnetic moment, Mz, along the main anisotropy axis for the studied systems. The green arrows correspond to the quantum tunneling mechanism of ground and first excited states, while the purple arrow shows the hypothetical Orbach relaxation process. The red arrow indicates the transition between the ground and first Kramers doublets. The values close to the arrows indicate the matrix elements of the transition magnetic moments (above 0.1, an efficient spin relaxation mechanism is expected).

High-level calculations including spin–orbit effects through the RASSI approach (see the Computational Details section) based on the CASSCF methodology have been employed to study the magnetic properties of the reported compounds. Cerium(III) complexes have 4f^1^ and ^2^F_5/2_ ground states. Magnetic susceptibility and magnetization have been simulated and show similar trends to those observed experimentally for compounds **1–4**, Figures S25 and S26. All the systems show an axial anisotropy with a relatively large *g*_*z*_ component consistent with an *m*_J_ = ±5/2 ground state, but the values reveal a contribution of *m*_J_ = ±3/2 states. The calculated *g* tensors are collected in Table 4, where the *g*_*z*_ values are in the range of 2.9–3.9, while for a perfectly axial Ce^III^ complex, the value is 4.19 (30/7). The calculated *g*_*z*_ orientations (see Figure 6) for the four compounds are quite different, and also, the CNs are 8 for **1** and **2**, 9 for **3**, and 10 for **4**. For an *m*_J_ = ±5/2 ground state, an oblate f electron density is expected with a perpendicular *g*_*z*_ orientation. Basically, there are two criteria that control the *g*_*z*_ orientation in order to reduce the metal–ligand electrostatic repulsion: (i) the alignment of *g*_*z*_ with the shortest Ce–O bond distances, so the f electron density disc remains perpendicular to that direction, and (ii) the f electron density disc is aligned in the same plane as that of the longest Ce–L bond distances, which are the Ce–N distances from pyridyl-type ligands or Ce–O distances involving MeOH in **1**. For **1**, *g*_*z*_ is approximately aligned with one of the shortest Ce–O bond distances (Ce1–O5), allowing the f electron density disc to remain in the plane where the coordinated methanol molecules are. In the case of **2**, the *g*_*z*_ is located in a way that the electron density disc avoids all the ligands. For **3**, there are two crystallographic independent molecules that differ mainly in the arrangement of one of the diketo anions. For **3b**, *g*_*z*_ is located in a way that the density disc is in the plane where the terpy is; however, for **3a**, the avoidance of the shortest Ce–O bond distances results in the disc being aligned in a not so predictive plane but still where the Ce–L distances are larger. In the case of **4**, however, it is clear that the density is located in the plane almost containing both pyridine ligands.

**Table 4:** Calculated *g* Tensor Components for the Ground Kramers Doublet at the CASSCF-RASSI Level for the Studied Systems

complex	*g*_*x*_	*g*_*y*_	*g*_*z*_
**1**	1.064	1.304	3.160
**2**	0.223	0.374	3.321
**3a**	0.850	1.433	3.098
**3b**	0.860	1.837	2.920
**4**	0.092	0.437	3.908

Calculated Kramers doublet energies and transition probabilities between the states are represented in Figure 6. The tunneling probability in the ground state shows the smallest values for compounds **2** and **4**. This fact agrees with the experimental evidence that **2** starts to show a signal at a zero field. However, for **4**, such a behavior is probably hindered by the shorter intermolecular Ce···Ce distances, as already pointed out. The calculated largest tunneling effect in **1** and **3** is also reflected in the experimental field dependence τ^–1^ curves. A stronger external field is required to suppress such an effect in comparison with **2** and **4** systems. As already remarked, the high first excited energies allow us to rule out the Orbach spin relaxation, taking into account the fact that the fitted barriers with such a mechanism give values of around 20–30 cm^–1^.

## Conclusions

This work describes four new Ce^III^ compounds that behave as field-induced SMMs and increase the scarce family of Ce^III^ SMMs. Three β-diketone ligands, nfta, coordinate the Ce^III^ ions in these compounds, leaving other coordination positions to the solvent or polypyridyl ligands. Depending on the employed coligands, the CN oscillates from 8 to 10, showing the flexibility of the system and the possibilities of tuning it with the appropriate election of the ligand. The magnetic properties are also remarkable, all the complexes show a field-induced SMM behavior with a very small applied field needed in order to observe the behavior. Additionally, for **2**, a signal starts to appear at the zero field, showing this coordination and election of ligands as a promising one to achieve zero-field Ce^III^ SMMs if the QTM is decreased more. The analysis of the dynamic susceptibility measurements also brings some insights into the relaxation processes involved in the spin relaxation of Ce^III^ SMMs. The examination of the dependence with the field at 2.5 K clearly shows three different areas in the curves where different processes predominate. At low fields, QTM is the dominating process and can be suppressed with the application of very small external fields. The intermediate area is better described by the Raman process, and in the high fields, the direct process predominates. The fitting of the dependence with the temperature was performed using the Raman and local-mode terms and without including the Orbach process because, as shown by the calculations, the first excited state is at a larger energy (250–300 cm^–1^) than that obtained from a fit with an Orbach process (around 10–20 cm^–1^). The results show different contributions of both terms for the complexes, but in all of them, the local-mode term, which is not included routinely yet, cannot be ruled out as it has an important contribution or it can even be the predominant relaxation process. Moreover, we are providing a different explanation for the very low effective energy barriers usually obtained for Ce^III^ SMMs. In addition, DFT results show the presence of vibrational modes with a large contribution of the lanthanide at an energy similar to the obtained ω value, which might be responsible for the spin relaxation. Multiconfigurational calculations, on the other hand, show the axial anisotropy of the complexes with a relatively large *g*_*z*_ component, which is oriented in a way that the perpendicular f electron density disc avoids the shortest Ce–O bond distances and is in general in the same plane as that of the longest Ce–L bond distances. The transition probabilities between states show large QTM in general being smaller for **2** and **4** and the relaxation through the Orbach process at more than 250 cm^–1^. Nevertheless, the comparison of the studied systems shows the slower relaxation for **4**, which is the system with a larger *g*_*z*_ component, the one showing a smaller QTM probability, and it also presents a large Ce···Ce distance and smaller dipolar interactions. However, the complex showing smaller QTM is compound **2**, which also has a larger Ce···Ce distance and smaller dipolar interactions. It is also worth noting that compound **1**, besides the larger QTM, shows slower relaxation than **2** when the external field is applied. Even though in general more examples of Ce^III^ SMMs are needed to further understand and be able to tune the spin relaxation of this type of compounds, through this study, we are increasing this limited family and providing new insights into the understanding of their spin relaxation.

## Supporting Information Available

Powder X-ray diffraction data, crystal packing, main noncovalent interactions found, dynamic magnetic measurements and fits, and additional computational results (calculated susceptibility and magnetization curves and Kramers doublet energies and g tensor components) (PDF)

## Accession Codes

CCDC 2100954–2100957 contain the supplementary crystallographic data for this paper. These data can be obtained free of charge via www.ccdc.cam.ac.uk/data_request/cif, or by emailing data_request@ccdc.cam.ac.uk, or by contacting The Cambridge Crystallographic Data Centre, 12 Union Road, Cambridge CB2 1EZ, UK; fax: +44 1223 336033.
