# Supplementary material for: Insights into the Spin Dynamics of Mononuclear Cerium(III) Single-Molecule Magnets
Source: Inorg Chem. 2022 Jul 11;61(29):11124–36. doi: 10.1021/acs.inorgchem.2c00958 (PMC9490812; doi:10.1021/acs.inorgchem.2c00958)
Supplement: Supplementary file 1 — ic2c00958_si_001.pdf [file ic2c00958_si_001.pdf]

Supporting Information

Insights into the Spin Dynamics in Mononuclear Cerium(III) Single-Molecule Magnets

Franz A. Mautner,[†] Florian Bierbaumer,[‡] Roland C. Fischer,[‡] Annia Tubau,[§] Saskia Speed,[§] Eliseo Ruiz,[§,¤] Salah S. Massoud,[ǂǂ] Ramon Vicente,[§] and Silvia Gómez-Coca[§,¤]*

† Institut für Physikalische und Theoretische Chemie, Technische Universität Graz, Stremayrgasse 9, A-8010 Graz, Austria

‡ Institut für Anorganische Chemie, Technische Universität Graz, Stremayrgasse 9, A-8010 Graz, Austria

§ Departament de Química Inorgànica i Orgànica, Universitat de Barcelona, Martí i Franquès 1-11, E-08028 Barcelona, Spain

¤ Institut de Recerca de Química Teòrica i Computacional, Universitat de Barcelona, Martí i Franquès 1-11, E-08028 Barcelona, Spain

ǂǂ Department of Chemistry, University of Louisiana at Lafayette, P.O. Box 43700, Lafayette, LA 70504, U.S.A.

Contents **page #**

1. Powder X-ray Diffraction Data ………………………………………. S2

2. Single-Crystal X-ray Diffraction Data ……………………………. S6

3. Non-covalent Bonding Interactions in Complexes **1-4** …………….. S8

4. Magnetic Data ………………………………………………………… S17

5. Theoretical Results …………………………………………………… S33

1. Powder X-ray Diffraction Data

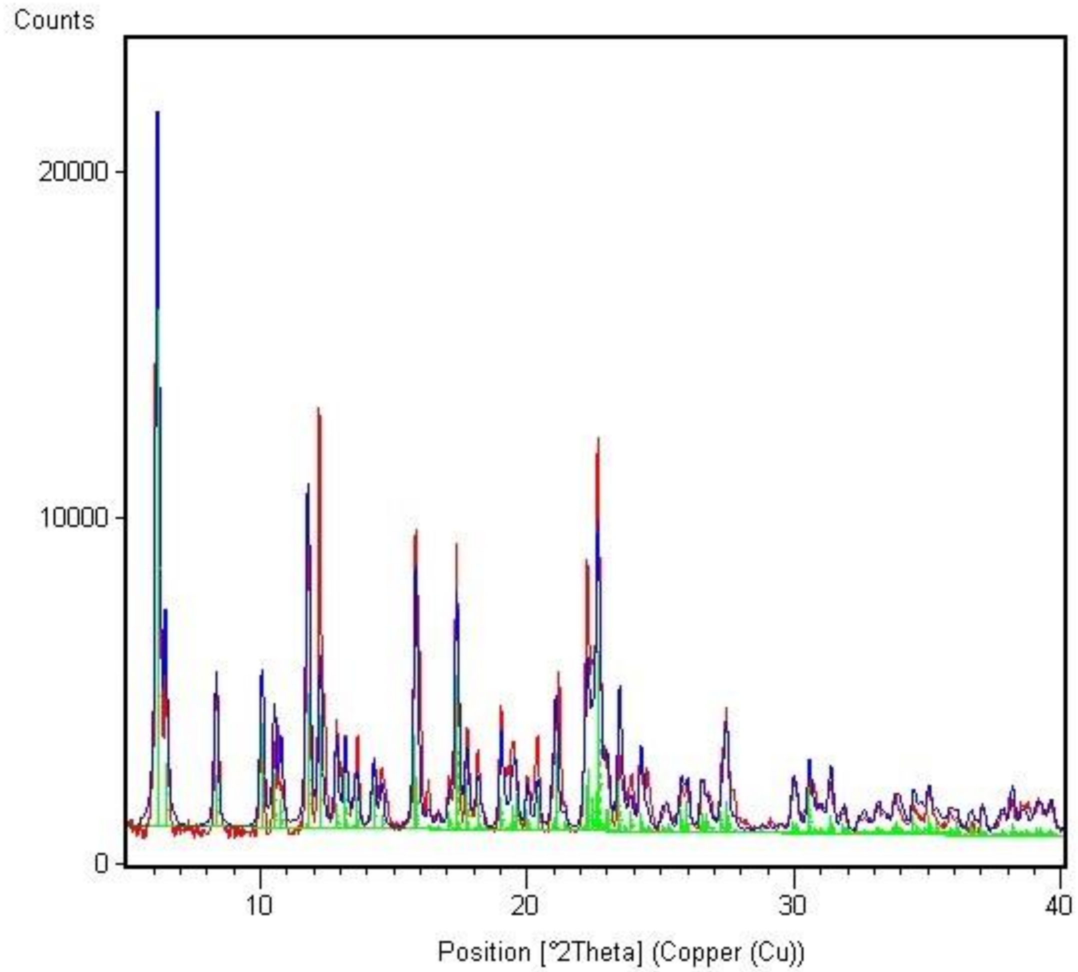

Figure S1. Observed and simulated PXRD of [Ce(ntfa)$_3$(MeOH)$_2$] (**1**).

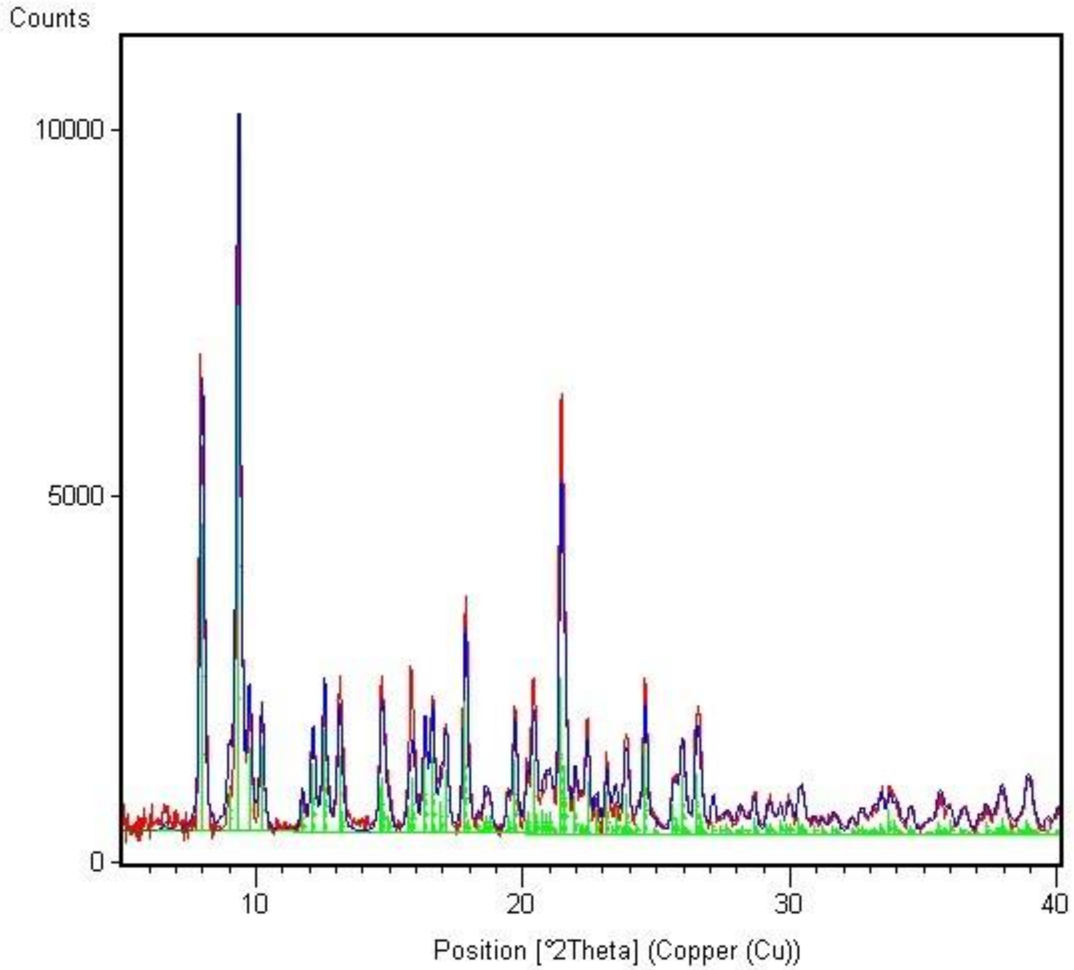

Figure S2. Observed and simulated PXRD of [Ce(ntfa)$_3$(5,5'-Me$_2$bipy)] (**2**).

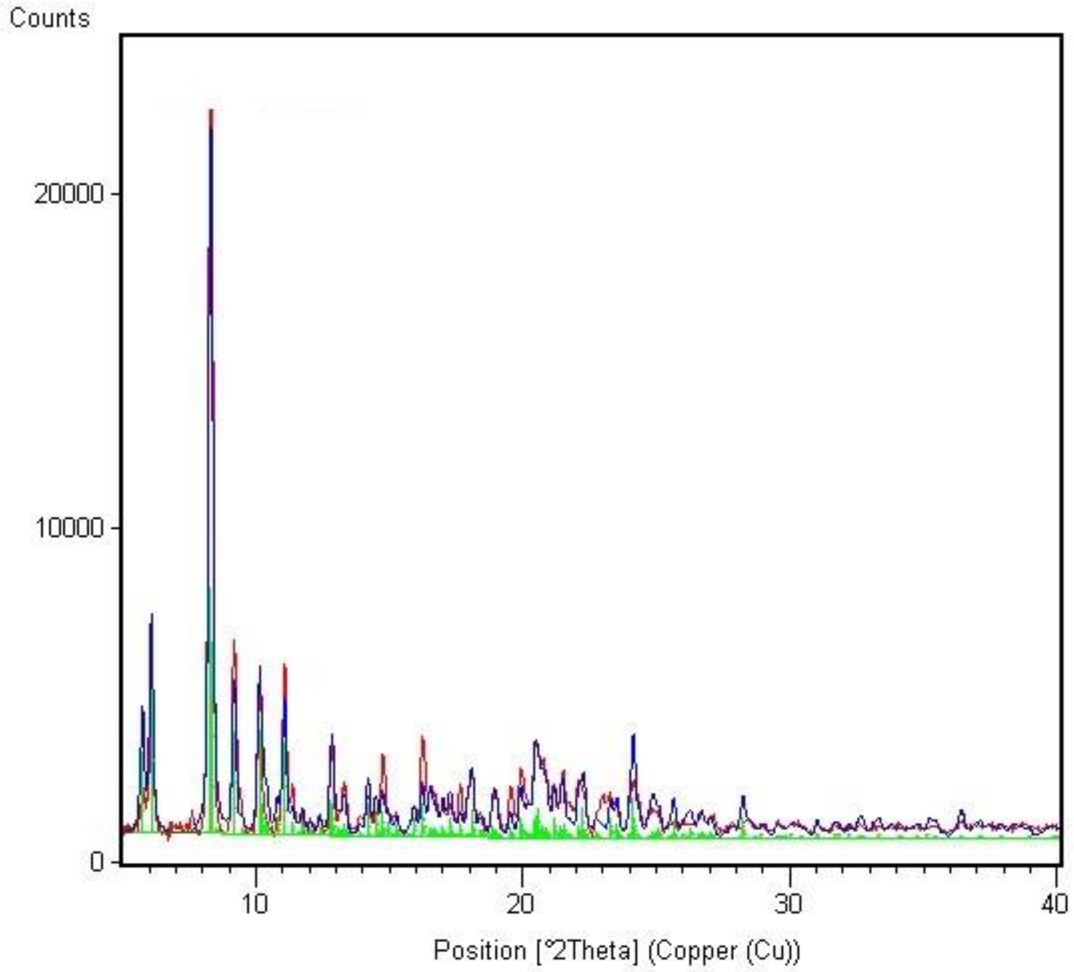

Figure S3. Observed and simulated PXRD of [Ce(ntfa)$_3$(terpy)] (**3**).

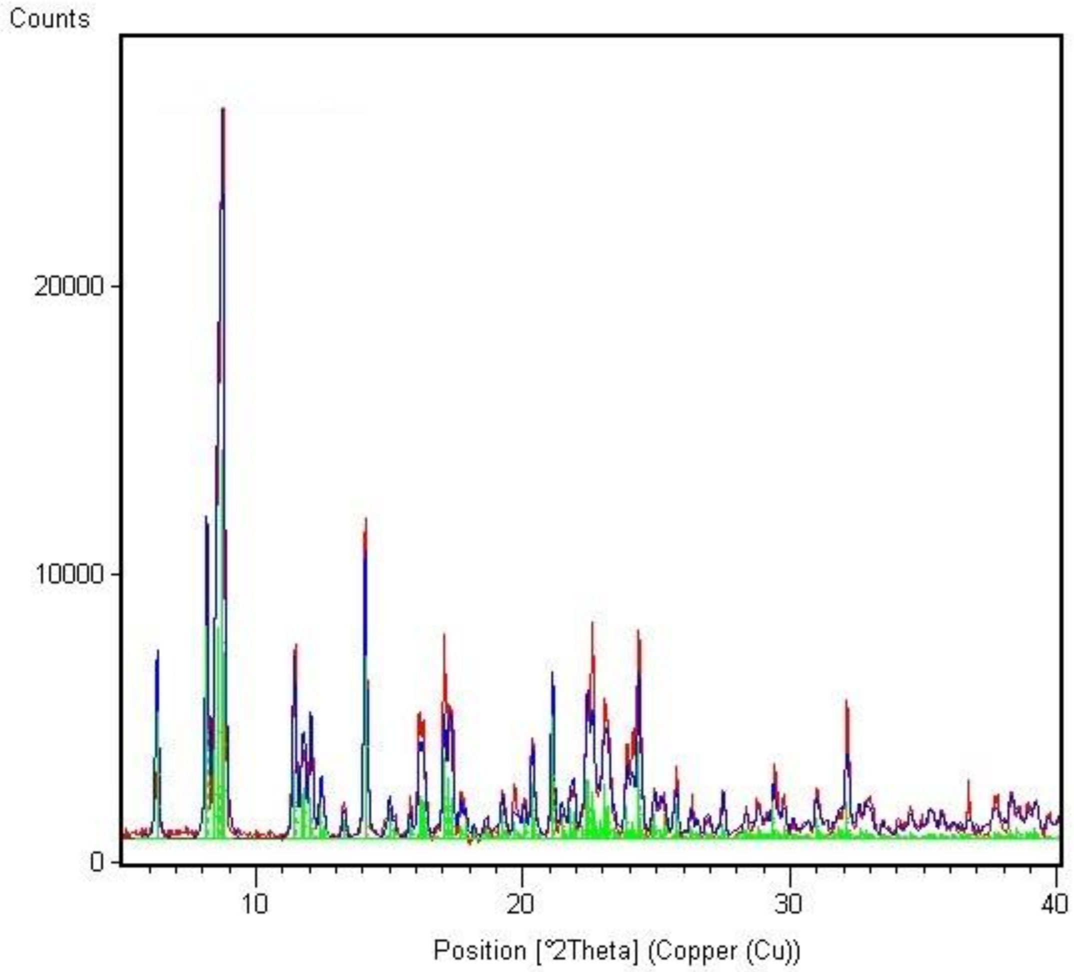

Figure S4. Observed and simulated PXRD of [Ce(ntfa)$_3$(bipy)$_2$] (**4**).

2. Single-Crystal X-ray Diffraction Data

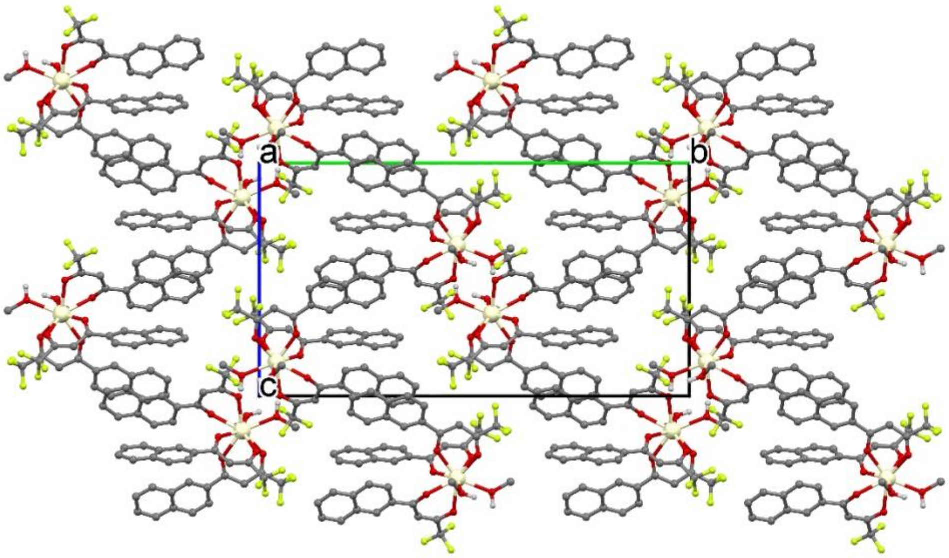

Figure S5. Crystal packing of **1**.

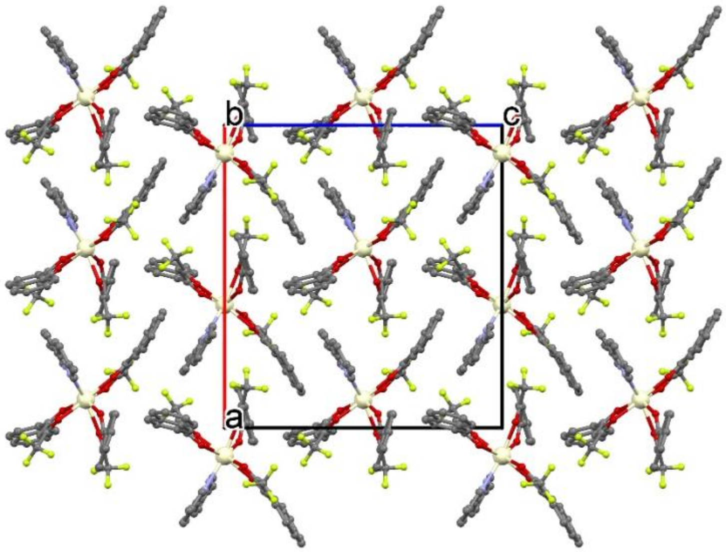

Figure S6. Crystal packing of **2**.

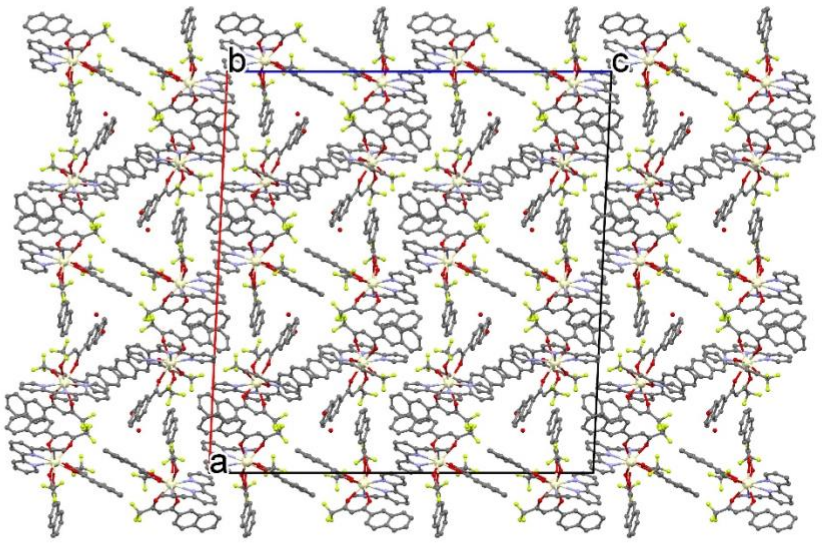

Figure S7. Crystal packing of **3**.

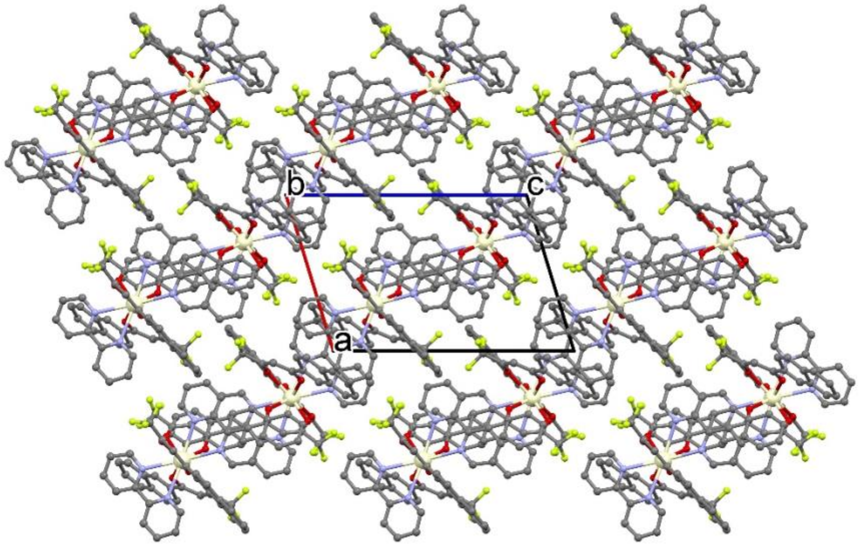

Figure S8. Crystal packing of **4**.

3. Non-covalent Bonding Interactions in Complexes 1 - 4

Table S1. Non-covalent interactions in **1**.

Definition of aromatic ring systems

6-Membered Ring (1) (C5 C7,C12-C14)

6-Membered Ring (2) (C7-C12)

6-Membered Ring (3) (C19-C21,C26-C28)

6-Membered Ring (4) (C21-C26)

6-Membered Ring (5) (C33-C35 C40 C42)

6-Membered Ring (6) (C35-C40)

10-Membered Ring (7) (C5-C14)

10-Membered Ring (8) (C19 C28)

10-Membered Ring (9) (C33 C42)

Analysis of Short Ring-Interactions.

- Cg(I) = Plane number I (= ring number in () above)
- Cg-Cg = Distance between ring Centroids (Ang.)

**Cg(I)**	**Cg(J)**	**[ARU(J)]**	**Cg-Cg**
Cg3	Cg6	[4555]	3,720(2)
Cg3	Cg9	[4555]	4,1699(19)
Cg4	Cg5	[4555]	3,755(2)
Cg4	Cg6	[4555]	3,989(2)
Cg4	Cg9	[4555]	3,6813(19)
Cg5	Cg8	[4554]	4,1821(19)
Cg6	Cg8	[4554]	3,6600(19)
Cg8	Cg9	[4555]	3,7399(17)

[4554]	X,1/2-Y,-1/2+Z

[4555]	X,1/2-Y,1/2+Z

Analysis of X-H...Cg(Pi-Ring) Interactions

- Cg(J) = Center of gravity of ring J (Plane number above)
- X-H..Cg = X-H-Cg angle (degrees)
- X..Cg = Distance of X to Cg (Angstrom)

X--H(I)	Cg(J)	[ARU(J)]	H..Cg	X-H..Cg	X..Cg
C8-H8	Cg3	[1555]	2,69	149	3,540(4)
C8-H8	Cg8	[1555]	2,82	137	3,575(4)
C13-H13	Cg6	[1655]	2,86	142	3,652(4)
C13-H13	Cg9	[1655]	2,67	135	3,405(4)
C22-H22	Cg1	[4555]	2,73	143	3,532(3)
C39-H39	Cg2	[4454]	2,9	143	3,703(3)
C39-H39	Cg7	[4454]	2,98	134	3,707(3)

[1555]	X,Y,Z
[1655]	1+X,Y,Z
[4555]	X,1/2-Y,1/2+Z
[4454]	-1+X,1/2-Y,-1/2+Z

Table S2. Non-covalent interactions in **2**.

Definition of aromatic ring systems

6-Membered Ring (1) (N1,C43-C47)

6-Membered Ring (2) (N2,C49-C53)

6-Membered Ring (3) (C5-C7,C12-C14)

6-Membered Ring (4) (C7- C12)

6-Membered Ring (5) (C19-C21,C26-C28)

6-Membered Ring (6) (C21-C26)

6-Membered Ring (7) (C33-C35,C40-C42)

6-Membered Ring (8) (C35-C40)

10-Membered Ring (9) (C5-C14)

10-Membered Ring (10) (C19-C28

10-Membered Ring (11) (C33-C42)

Analysis of Short Ring-Interactions

- Cg(I) = Plane number I (= ring number in () above)

- Cg-Cg = Distance between ring Centroids (Ang.)

Cg(I)	Cg(J)	[ARU(J)]	Cg-Cg
Cg2	Cg3	[3554]	3,865(2)
Cg2	Cg4	[3554]	3,805(2)
Cg2	Cg9	[3554]	3,638(2)
Cg3	Cg6	[4475]	4,214(2)
Cg3	Cg10	[4475]	4,1754(18)

[3554]	1/2-X,Y,-1/2+Z
[4475]	-1/2+X,2-Y,Z

Analysis of X-H...Cg(Pi-Ring) Interactions

- Cg(J) = Center of gravity of ring J (Plane number above)
- X-H..Cg = X-H-Cg angle (degrees)
- X..Cg = Distance of X to Cg (Angstrom)

**X--H(I)**	**Cg(J)**	**[ARU(J)]**	**H..Cg**	**X-H..Cg**	**X,,Cg**
C10-H10	Cg8	[3565]	2,79	143	3,598(4)
C36-H36	Cg5	[1545]	2,95	142	3,739(4)
C36-H36	Cg6	[1545]	2,9	130	3,585(4)
C36-H36	Cg10	[1545]	2,65	142	3,446(4)
C44-H44	Cg8	[4465]	2,83	152	3,702(4)
C44-H44	Cg11	[4465]	2,89	132	3,597(4)
C45-H45	Cg7	[4465]	2,88	137	3,636(4)

[3565]	1/2-X,1+Y,1/2+Z
[1545]	X,-1+Y,Z
[4465]	-1/2+X,1-Y,Z

Table S3. Non-covalent interactions in **3**.

Definition of aromatic ring systems

```
  6-Membered Ring ( 1)  (N4,C100-C104)
  6-Membered Ring ( 2)  (N5,C105-C109)
  6-Membered Ring ( 3)  (N6,C110-C114)
  6-Membered Ring ( 4)  (C62-C64,C69-C71)
  6-Membered Ring ( 5)  (C64-C69
  6-Membered Ring ( 6)  (C76-C78,C83-C85)
  6-Membered Ring ( 7)  (C78-C83)
  6-Membered Ring ( 8)  (C90-C92,C97-C99)
  6-Membered Ring ( 9)  (C92-C97)
  6-Membered Ring (10)  (N1,C43-C47)
  6-Membered Ring (11)  (N2,C48-C52)
  6-Membered Ring (12)  (N3,C53-C57)
  6-Membered Ring (13)  (C5-C7,C12-C14)
  6-Membered Ring (14)  (C7-C12)
  6-Membered Ring (15)  (C19-C21,C26-C28)
  6-Membered Ring (16)  (C21-C26)
  6-Membered Ring (17)  (C33-C42)
  6-Membered Ring (18)  (C35-C40)
```

Short Ring-Ring Interactions

```
- Cg(I)    = Plane number I (= ring number in () above)
- Cg-Cg    = Distance between ring Centroids (Ang.)

Cg(I) Cg(J)   [ARU(J)]    Cg-Cg

Cg2    Cg3    [3666]    3.657(5)
Cg2    Cg13   [5555]    4.326(4)
```

Cg4	Cg17	[1555]	4.066(5)
Cg5	Cg17	[1555]	3.629(6)
Cg5	Cg18	[1555]	3.786(6)
Cg6	Cg6	[2655]	3.656(7)
Cg6	Cg7	[2655]	4.215(7)
Cg7	Cg7	[2655]	3.798(7)
Cg12	Cg16	[1565]	3.659(4)

[1565] = X,1+Y,Z

[5555] = 1/2+X,1/2+Y,Z

[3666] = 1-X,1-Y,1-Z

[1555] = X,Y,Z

[2655] = 1-X,Y,1/2-Z

[1565] = X,1+Y,Z

Analysis of X-H...Cg(Pi-Ring) Interactions

- Cg(J) = Center of gravity of ring J (Plane number above)
- X-H..Cg = X-H-Cg angle (degrees)
- X..Cg = Distance of X to Cg (Angstrom)

X--H(I)		Cg(J)	[ARU(J)]	H..Cg	X-H..Cg	X..Cg
C25	-H25	Cg17	[6545]	2.98	147	3.805(8)
C46	-H46	Cg14	[1565]	2.68	142	3.478(10)
C49	-H49	Cg13	[1565]	2.78 -	146	3.608(7)
C51	-H51	Cg15	[1565]	2.83	120	3.405(7)
C55	-H55	Cg18	[6555]	2.59	165	3.515(9)
C56	-H56	Cg16	[6555]	2.84	141	3.623(8)
C91	-H91	Cg3	[1555]	2.94	149	3.782(8)
C106	-H106	Cg9	[1565]	2.95	174	3.891(12)

```
C107   -H107 Cg4    [3666]        2.97          142   3.759(13)


[6545] = 1/2-X,-1/2+Y,1/2-Z

[1565] = X,1+Y,Z

[6555] = 1/2-X,1/2+Y,1/2-Z

[1555] = X,Y,Z

[1565] = X,1+Y,Z

[3666] = 1-X,1-Y,1-Z
```

Analysis of Y-F...Cg(Pi-Ring) Interactions

Y--F(I)		Cg(J)	F..Cg	Y-F..Cg	Y..Cg
C1	-F3	Cg12	3.425(6)	116.0(4)	4.197(8)
C58	-F12	Cg1	3.792(9)	102.0(7)	4.256(14)
C29A	-F8A	Cg11	3.723(18)	134.5(10)	4.757(14)

Table S4. Non-covalent interactions in **4**.

Definition of aromatic ring systems

```
  6-Membered Ring ( 1)  (N1-C43-C47)

  6-Membered Ring ( 2)  (N2,C48-C52)

  6-Membered Ring ( 3)  (N3,C53-C57)

  6-Membered Ring ( 4)  (N4,C58-C62)

  6-Membered Ring ( 5)  (C5-C7,C12-C14)

  6-Membered Ring ( 6)  (C7-C12)

  6-Membered Ring ( 7)  (C19-C21,C26-C28)

  6-Membered Ring ( 8)  (C21-C26)

  6-Membered Ring ( 9)  (C33-C35,C40-C42)

  6-Membered Ring (10)  (C35-C40)
```

Ring-Interactions with Cg-Cg Distances

```
- Cg(I)    = Plane number I (= ring number in () above)

- Cg-Cg    = Distance between ring Centroids (Ang.)


Cg(I)  Cg(J)  [ARU(J)]  Cg-Cg


Cg1    Cg1    [2567]    4.216(3)

Cg1    Cg2    [2567]    4.024(2)

Cg3    Cg3    [2666]    3.750(3)

Cg7    Cg7    [2656]    4.037(3)

Cg9    Cg9    [2557]    3.824(3)

Cg9    Cg10   [2557]    3.725(3)


[2567] = -X,1-Y,2-Z;  [2557] = -X,-Y,2-Z;  [2666] = 1-X,1-Y,1-Z
```

Analysis of X-H...Cg(Pi-Ring) Interactions

```
- Cg(J)   = Center of gravity of ring J (Plane number above)

- X-H..Cg = X-H-Cg angle (degrees)
```

- X..Cg = Distance of X to Cg (Angstrom)

X--H(I)		Cg(J)	[ARU(J)]	H..Cg	X-H..Cg	X..Cg
C24	-H24	Cg10	[2656]	2.98	137	3.738(6)
C25	-H25	Cg9	[2656]	2.72	148	3.567(5)
C59	-H59	Cg5	[2666]	2.72	151	3.582(5)
C60	-H60	Cg6	[2666]	2.74	147	3.576(5)

[2656] = 1-X,-Y,1-Z; [2666] = 1-X,1-Y,1-Z

Analysis of Y-F...Cg(Pi-Ring) Interactions

Y--X(I)		Cg(J)	[ARU(J)]	F..Cg	Y-F..Cg	Y..Cg
C15	-F6	Cg6	[1645]	3.610(4)	128.8(3)	4.569(5)
C29	-F8A	Cg4	[1455]	3.946(19)	124.9(10)	4.757(11)

[1455] = -1+X,Y,Z; [1645] = 1+X,-1+Y,Z

4. Magnetic Data

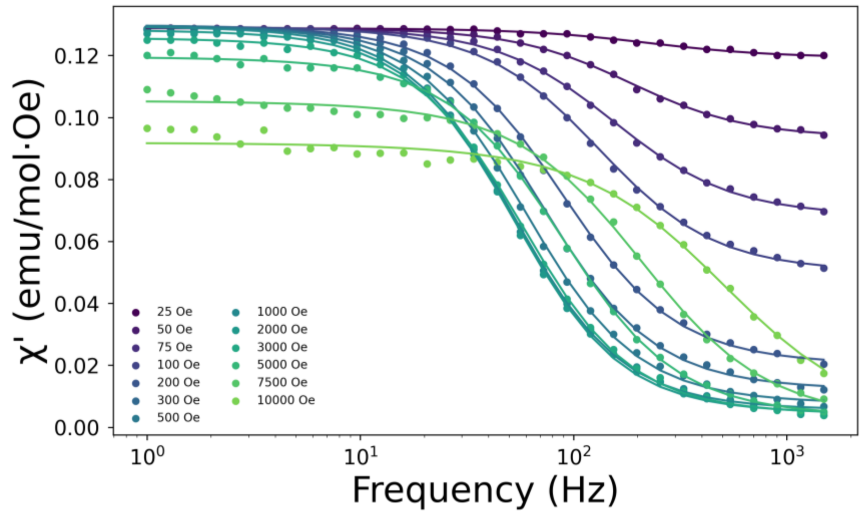

Figure S9. Field and frequency dependence of the in phase (χ'_M) component of the ac susceptibility for compound **1** acquired at 2.5 K

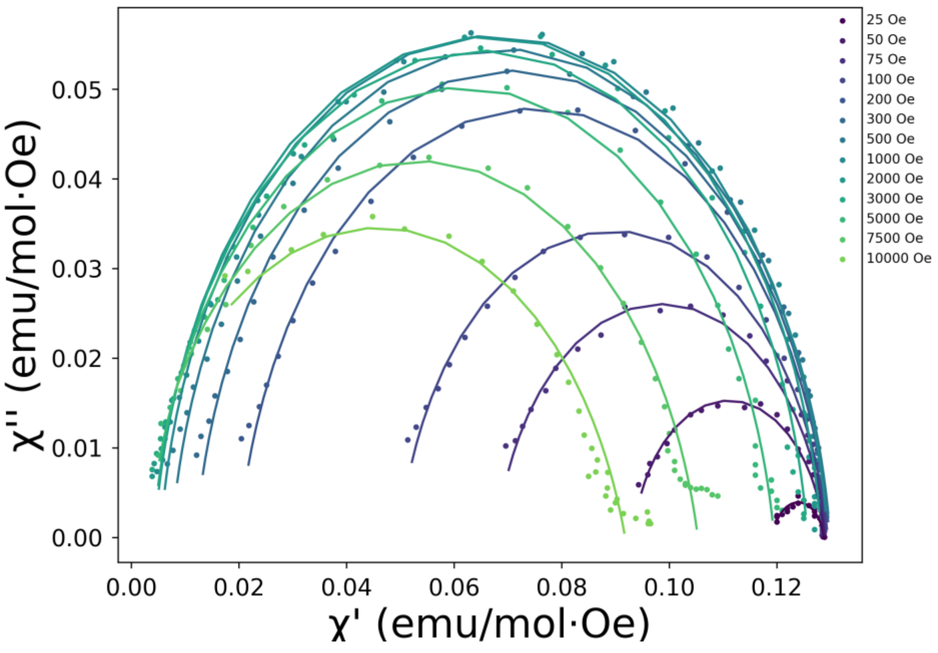

Figure S10. Cole-cole plot for compound **1** acquired at 2.5 K and different fields. Lines are the result of the fit of the data to a Debye function using the CCfit package.

Table S5. Results from the fit of Cole-Cole plots to a Debye function using the CCfit package for **1** at 2.5 K and different applied dc fields.

Field (Oe)	χ_S	χ_t	τ (s)	α	residual
25	1.19E-01	1.29E-01	7.08E-04	1.26E-01	4.01E-06
50	9.33E-02	1.29E-01	8.62E-04	9.82E-02	9.60E-06
75	6.79E-02	1.29E-01	1.03E-03	1.05E-01	2.96E-05
100	4.99E-02	1.30E-01	1.21E-03	9.67E-02	3.58E-05
200	2.00E-02	1.30E-01	1.78E-03	8.74E-02	5.55E-05
300	1.20E-02	1.30E-01	2.20E-03	7.91E-02	5.73E-05
500	7.49E-03	1.30E-01	2.63E-03	7.41E-02	6.67E-05
1000	5.43E-03	1.30E-01	2.99E-03	6.55E-02	6.20E-05
2000	4.31E-03	1.28E-01	2.99E-03	6.59E-02	5.28E-05
3000	4.05E-03	1.26E-01	2.75E-03	7.23E-02	4.67E-05
5000	3.29E-03	1.20E-01	1.81E-03	9.35E-02	4.57E-05
7500	1.73E-03	1.05E-01	7.63E-04	1.33E-01	1.48E-04
10000	2.72E-07	9.18E-02	3.14E-04	1.79E-01	2.01E-04

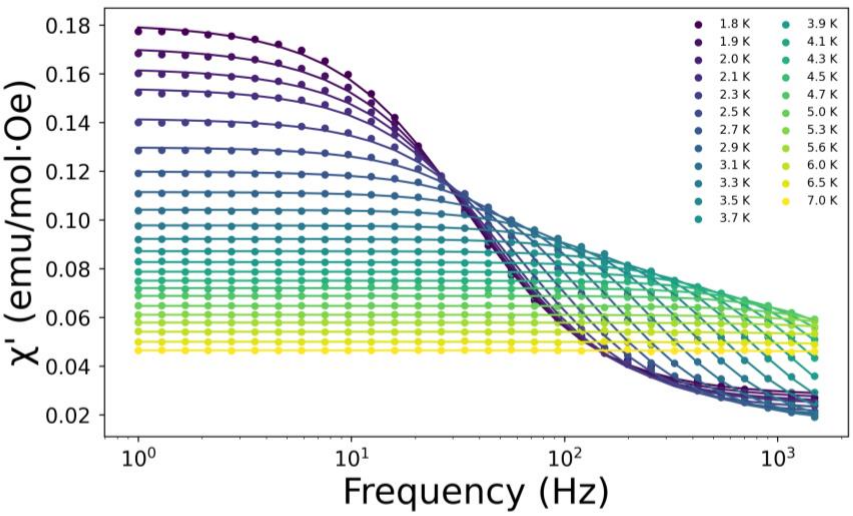

Figure S11. Temperature and frequency dependence of the in phase (χ'_M) component of the ac susceptibility for compound **1** acquired at 200 Oe.

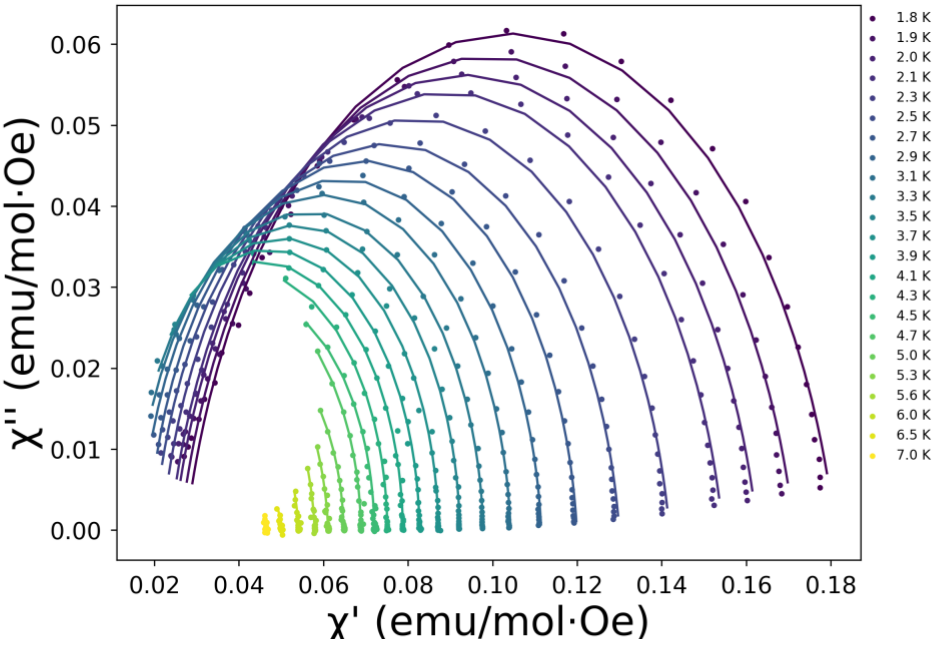

Figure S12. Cole-cole plot for compound **1** acquired at 200 Oe and different temperatures. Lines are the result of the fit of the data to a Debye function using the CCfit package.

Table S6. Results from the fit of Cole-Cole plots to a Debye function using the CCfit package for **1** at 200 Oe applied dc field and different temperatures.

T (K)	χ_S	χ_t	τ (s)	α	residual
1.8	2.74E-02	1.81E-01	4.63E-03	1.42E-01	1.38E-04
1.9	2.61E-02	1.71E-01	4.09E-03	1.37E-01	1.23E-04
2	2.46E-02	1.63E-01	3.59E-03	1.30E-01	9.89E-05
2.1	2.37E-02	1.54E-01	3.15E-03	1.21E-01	8.87E-05
2.3	2.18E-02	1.42E-01	2.45E-03	1.06E-01	8.11E-05
2.5	2.00E-02	1.30E-01	1.78E-03	9.03E-02	5.41E-05
2.69	1.87E-02	1.20E-01	1.29E-03	6.60E-02	3.74E-05
2.9	1.70E-02	1.12E-01	8.99E-04	5.54E-02	2.49E-05
3.1	1.57E-02	1.04E-01	6.21E-04	4.34E-02	1.66E-05
3.3	1.47E-02	9.78E-02	4.31E-04	3.57E-02	7.59E-06
3.5	1.44E-02	9.23E-02	3.05E-04	2.17E-02	8.62E-06
3.71	1.33E-02	8.72E-02	2.15E-04	1.57E-02	6.02E-06
3.9	1.15E-02	8.27E-02	1.52E-04	1.72E-02	3.68E-06
4.11	1.11E-02	7.88E-02	1.12E-04	1.27E-02	3.50E-06
4.31	1.04E-02	7.51E-02	8.26E-05	1.11E-02	2.54E-06
4.51	1.35E-02	7.20E-02	6.50E-05	1.11E-02	4.18E-06
4.7	8.01E-03	6.89E-02	4.58E-05	1.79E-02	3.20E-06
5	1.54E-02	6.48E-02	3.57E-05	2.04E-02	1.77E-06
5.3	1.96E-09	6.11E-02	1.75E-05	5.12E-02	2.75E-06
5.6	8.58E-10	5.79E-02	1.27E-05	4.97E-02	3.05E-06
6	1.17E-09	5.42E-02	7.88E-06	7.92E-02	1.57E-06
6.5	1.78E-09	5.00E-02	3.48E-06	1.49E-01	3.06E-06
7	3.41E-21	4.65E-02	2.36E-06	1.29E-01	1.93E-06

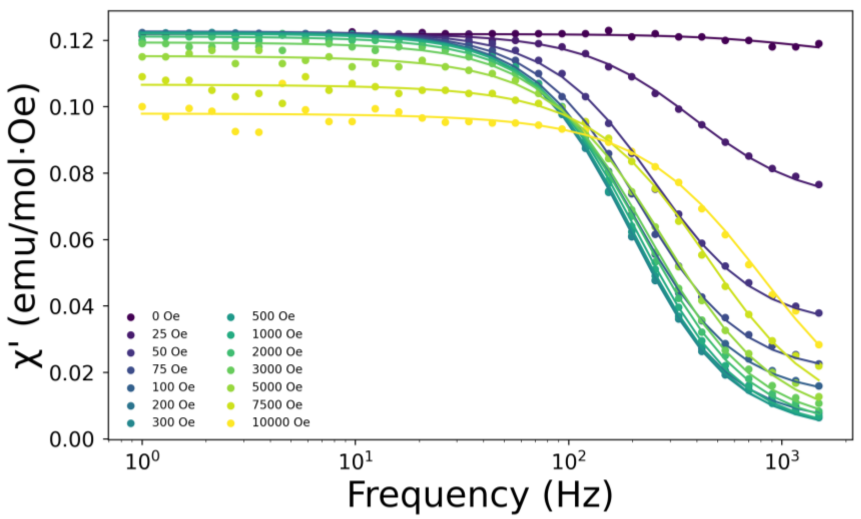

Figure S13. Field and frequency dependence of the in phase (χ'M) component of the ac susceptibility for compound **2** acquired at 2.5 K

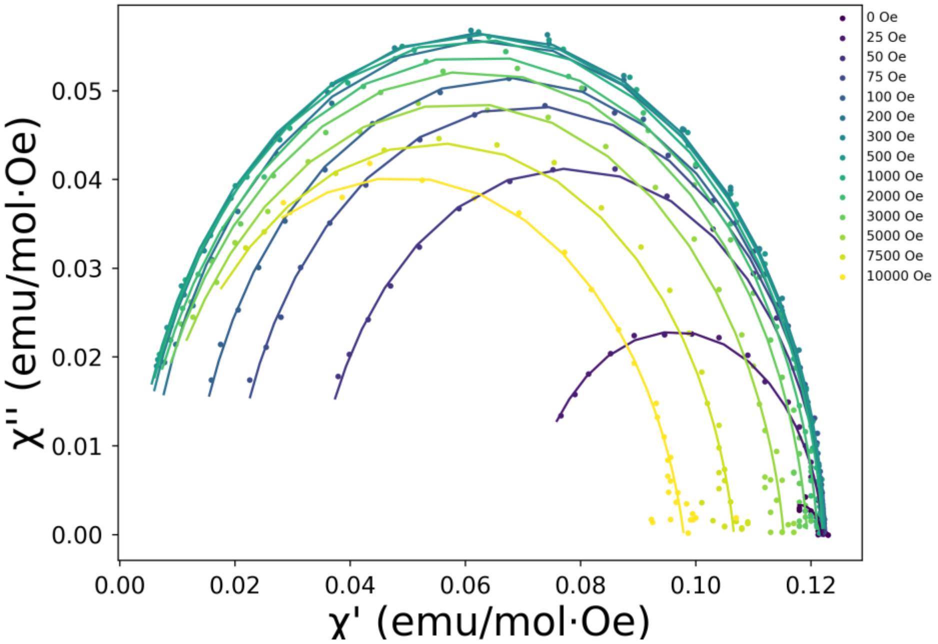

Figure S14. Cole-cole plot for compound **2** acquired at 2.5 K and different fields. Lines are the result of the fit of the data to a Debye function using the CCfit package.

Table S7. Results from the fit of Cole-Cole plots to a Debye function using the CCfit package for **2** at 2.5 K and different applied dc fields.

Field (Oe)	χ_S	χ_t	τ (s)	α	residual
0	1.14E-01	1.22E-01	1.14E-04	9.54E-02	1.25E-05
25	7.06E-02	1.22E-01	4.12E-04	7.74E-02	8.97E-06
50	3.34E-02	1.23E-01	6.39E-04	5.10E-02	2.57E-05
75	1.92E-02	1.23E-01	7.43E-04	4.44E-02	2.37E-05
100	1.21E-02	1.23E-01	7.89E-04	4.54E-02	1.78E-05
200	4.57E-03	1.22E-01	8.28E-04	3.65E-02	2.78E-05
300	2.83E-03	1.22E-01	8.16E-04	3.66E-02	2.60E-05
500	1.96E-03	1.22E-01	7.89E-04	4.27E-02	3.30E-05
1000	2.04E-03	1.22E-01	7.56E-04	4.71E-02	3.03E-05
2000	2.50E-03	1.21E-01	7.15E-04	6.11E-02	9.09E-05
3000	3.36E-03	1.19E-01	6.72E-04	6.76E-02	9.43E-05
5000	3.75E-03	1.15E-01	5.61E-04	8.78E-02	7.12E-05
7500	4.15E-03	1.07E-01	3.63E-04	9.61E-02	1.85E-04
10000	1.26E-12	9.79E-02	1.98E-04	1.25E-01	2.74E-04

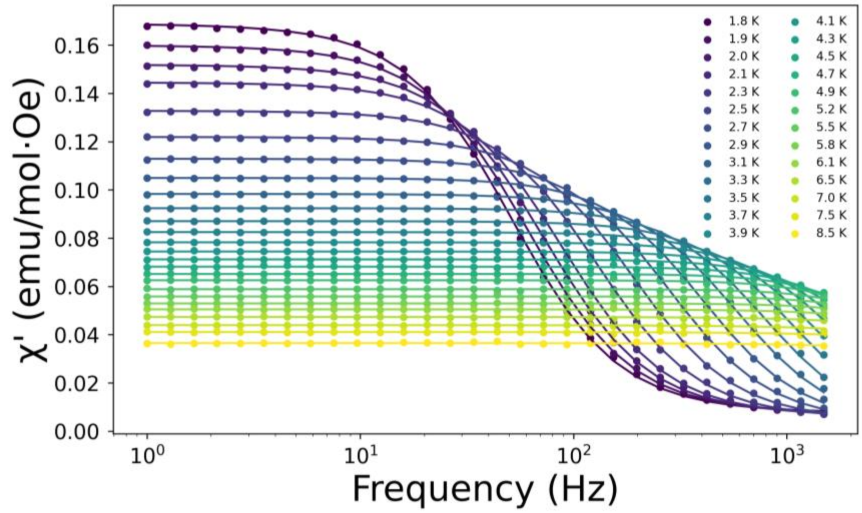

Figure S15. Temperature and frequency dependence of the in phase (χ'_M) component of the ac susceptibility for compound **2** acquired at 200 Oe.

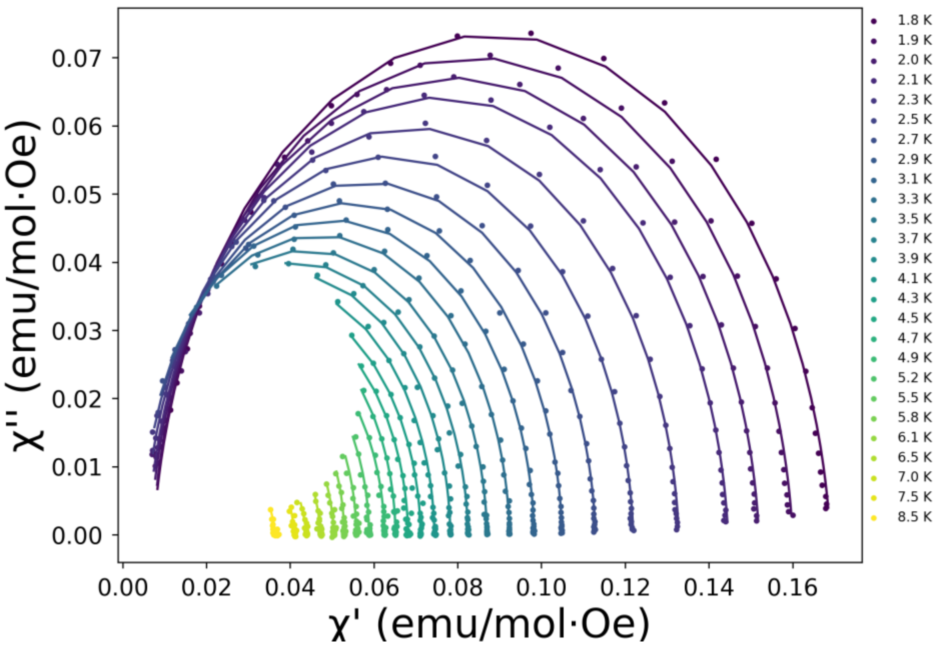

Figure S16. Cole-cole plot for compound **2** acquired at 200 Oe and different temperatures. Lines are the result of the fit of the data to a Debye function using the CCfit package.

Table S8. Results from the fit of Cole-Cole plots to a Debye function using the CCfit package for **2** at 200 Oe applied dc field and different temperatures.

T (K)	χ_S	χ_t	τ (s)	α	residual
1.8	7.38E-03	1.69E-01	3.11E-03	6.14E-02	7.11E-05
1.89	7.24E-03	1.60E-01	2.62E-03	5.52E-02	5.44E-05
2	6.66E-03	1.52E-01	2.16E-03	5.10E-02	5.35E-05
2.1	6.08E-03	1.45E-01	1.77E-03	4.87E-02	4.06E-05
2.3	5.30E-03	1.33E-01	1.23E-03	4.21E-02	3.49E-05
2.49	4.49E-03	1.22E-01	8.25E-04	3.61E-02	2.07E-05
2.7	3.84E-03	1.13E-01	5.64E-04	3.38E-02	1.66E-05
2.9	3.17E-03	1.05E-01	3.92E-04	2.85E-02	1.10E-05
3.1	1.66E-03	9.83E-02	2.75E-04	2.93E-02	9.64E-06
3.3	1.59E-03	9.24E-02	2.03E-04	2.12E-02	6.89E-06
3.51	6.05E-04	8.70E-02	1.49E-04	2.24E-02	3.93E-06
3.7	6.18E-12	8.25E-02	1.13E-04	2.10E-02	5.54E-06
3.9	3.49E-12	7.83E-02	8.88E-05	1.26E-02	6.94E-06
4.11	3.92E-12	7.44E-02	7.13E-05	1.12E-02	3.52E-06
4.31	4.95E-12	7.12E-02	5.73E-05	1.72E-02	4.51E-06
4.5	4.03E-12	6.82E-02	4.73E-05	1.46E-02	4.67E-06
4.7	1.22E-11	6.51E-02	3.97E-05	5.39E-03	3.09E-06
4.9	1.38E-25	6.25E-02	3.35E-05	1.41E-02	2.88E-06
5.2	4.19E-25	5.88E-02	2.78E-05	3.04E-03	3.83E-06
5.5	2.32E-37	5.58E-02	2.29E-05	5.50E-03	3.50E-06
5.8	2.88E-48	5.29E-02	1.91E-05	1.09E-02	2.24E-06
6.1	2.30E-48	5.05E-02	1.52E-05	4.63E-02	3.30E-06
6.5	7.82E-49	4.73E-02	1.21E-05	7.44E-02	2.71E-06
7	8.43E-49	4.39E-02	9.00E-06	1.19E-01	3.09E-06
7.5	4.73E-49	4.11E-02	9.03E-06	9.24E-02	3.25E-06
8.5	4.21E-49	3.65E-02	7.25E-06	1.27E-01	4.60E-06

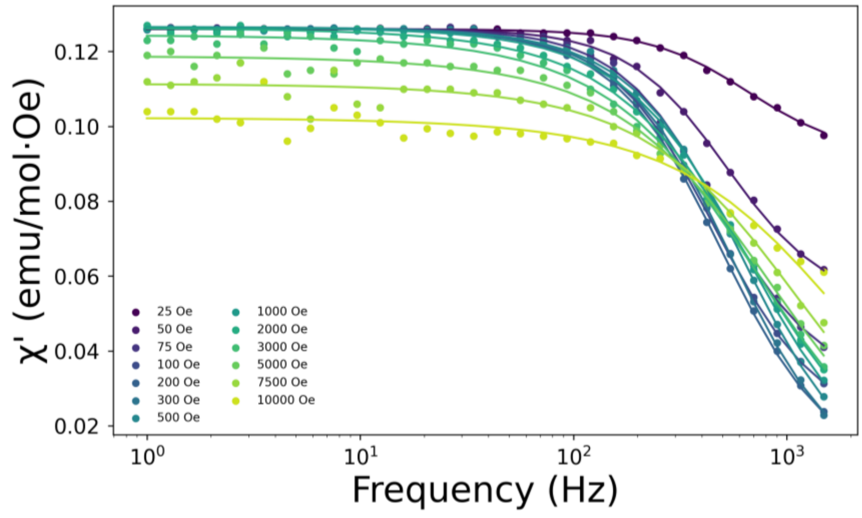

Figure S17. Field and frequency dependence of the in phase (χ'M) component of the ac susceptibility for compound **3** acquired at 2.5 K

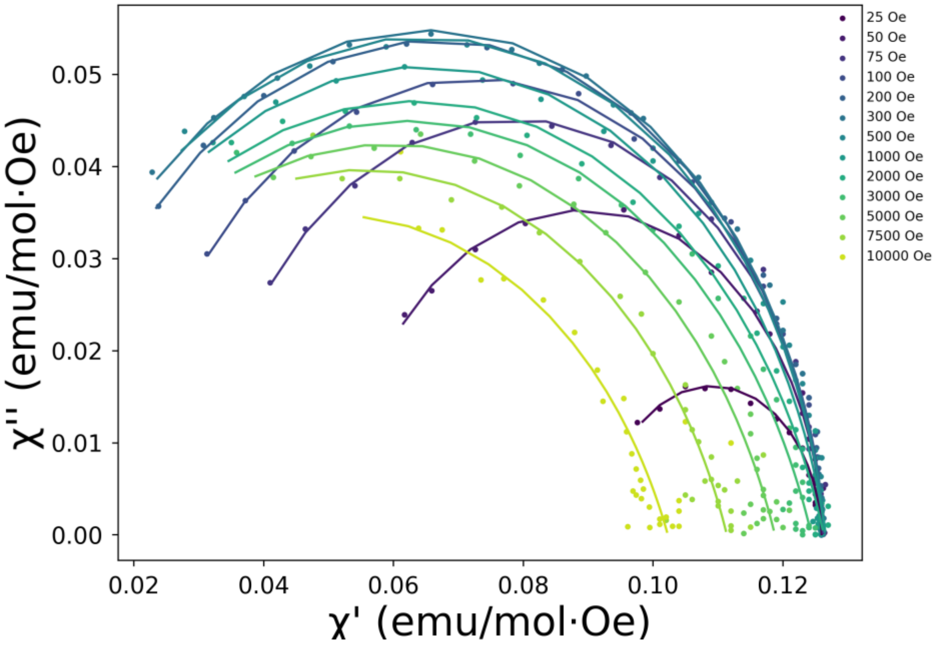

Figure S18. Cole-cole plot for compound **3** acquired at 2.5 K and different fields. Lines are the result of the fit of the data to a Debye function using the CCfit package.

Table S9. Results from the fit of Cole-Cole plots to a Debye function using the CCfit package for **3** at 2.5 K and different applied dc fields.

Field (Oe)	χ_S	χ_t	τ (s)	α	residual
25	9.23E-02	1.26E-01	2.40E-04	2.62E-02	6.34E-06
50	5.21E-02	1.26E-01	3.06E-04	3.18E-02	1.59E-05
75	3.08E-02	1.26E-01	3.36E-04	3.59E-02	1.67E-05
100	1.90E-02	1.26E-01	3.44E-04	5.10E-02	1.54E-05
200	6.77E-03	1.27E-01	3.20E-04	6.91E-02	1.71E-05
300	3.92E-03	1.26E-01	2.90E-04	7.12E-02	1.69E-05
500	2.74E-03	1.27E-01	2.56E-04	8.63E-02	3.44E-05
1000	2.49E-03	1.27E-01	2.42E-04	1.26E-01	4.03E-05
2000	1.50E-13	1.26E-01	2.31E-04	1.84E-01	7.87E-05
3000	1.66E-13	1.24E-01	2.28E-04	2.04E-01	9.12E-05
5000	2.45E-13	1.19E-01	1.97E-04	2.11E-01	1.24E-04
7500	3.84E-13	1.11E-01	1.48E-04	2.12E-01	3.78E-04
10000	5.97E-13	1.02E-01	9.17E-05	2.42E-01	5.41E-04

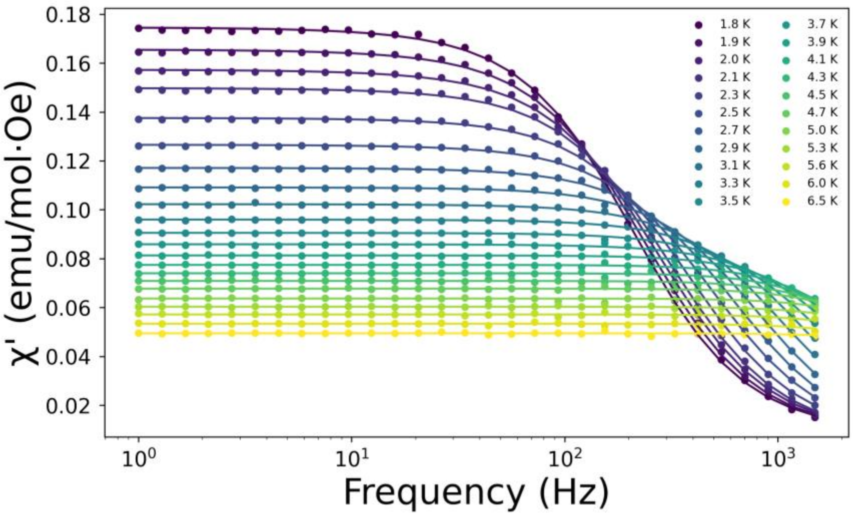

Figure S19. Temperature and frequency dependence of the in phase (χ'_M) component of the ac susceptibility for compound **3** acquired at 200 Oe.

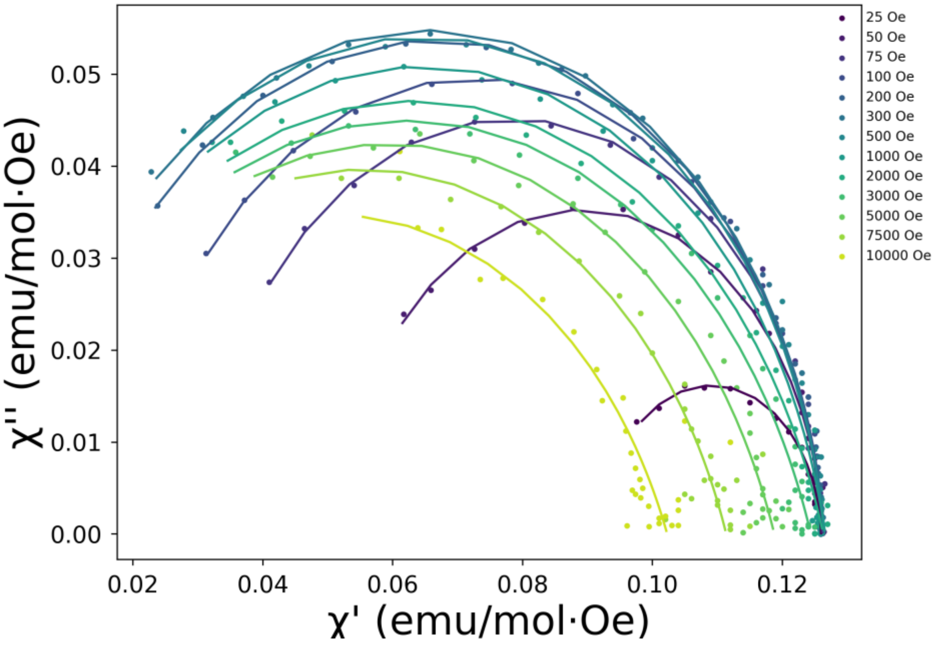

Figure S20. Cole-cole plot for compound **3** acquired at 200 Oe and different temperatures. Lines are the result of the fit of the data to a Debye function using the CCfit package.

Table S10. Results from the fit of Cole-Cole plots to a Debye function using the CCfit package for **3** at 200 Oe applied dc field and different temperatures.

T (K)	χ_S	χ_t	τ (s)	α	residual
1.79	8.23E-03	1.75E-01	7.62E-04	8.30E-02	4.30E-05
1.9	7.85E-03	1.66E-01	6.69E-04	8.01E-02	4.29E-05
2	7.34E-03	1.57E-01	5.84E-04	8.10E-02	3.11E-05
2.1	6.96E-03	1.50E-01	5.13E-04	7.74E-02	3.21E-05
2.3	7.42E-03	1.38E-01	4.13E-04	6.75E-02	2.12E-05
2.5	6.94E-03	1.27E-01	3.22E-04	6.71E-02	1.62E-05
2.7	7.01E-03	1.17E-01	2.53E-04	5.91E-02	1.12E-05
2.9	7.31E-03	1.09E-01	1.98E-04	4.96E-02	1.00E-05
3.1	7.84E-03	1.02E-01	1.56E-04	5.23E-02	1.49E-05
3.3	5.50E-03	9.60E-02	1.19E-04	4.80E-02	7.34E-06
3.52	8.08E-03	9.05E-02	9.69E-05	4.44E-02	1.07E-05
3.7	4.92E-03	8.59E-02	7.32E-05	4.77E-02	9.41E-06
3.9	1.16E-02	8.13E-02	6.70E-05	8.60E-03	5.65E-06
4.1	1.50E-02	7.74E-02	5.69E-05	5.73E-03	9.83E-06
4.31	1.73E-02	7.40E-02	5.10E-05	5.31E-17	9.73E-06
4.5	2.48E-02	7.09E-02	4.81E-05	4.90E-17	1.04E-05
4.7	2.18E-02	6.77E-02	3.80E-05	6.98E-17	7.24E-06
5	1.99E-02	6.37E-02	2.94E-05	8.60E-17	6.74E-06
5.3	3.63E-02	6.03E-02	3.73E-05	9.95E-17	4.07E-06
5.6	4.22E-02	5.71E-02	4.68E-05	9.92E-17	6.58E-06
6	4.34E-02	5.34E-02	5.30E-05	1.39E-16	6.78E-06
6.5	4.16E-02	4.94E-02	3.53E-05	4.66E-17	7.28E-06

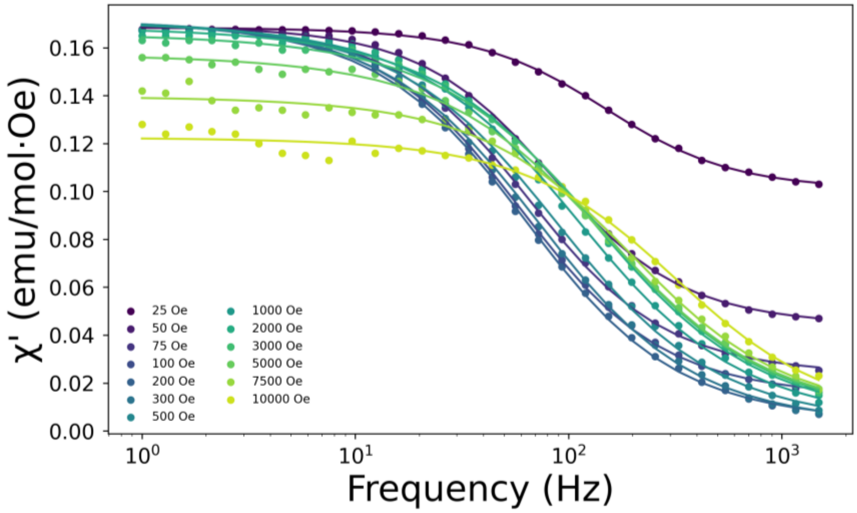

Figure S21. Field and frequency dependence of the in phase (χ'M) component of the ac susceptibility for compound **4** acquired at 2.5 K

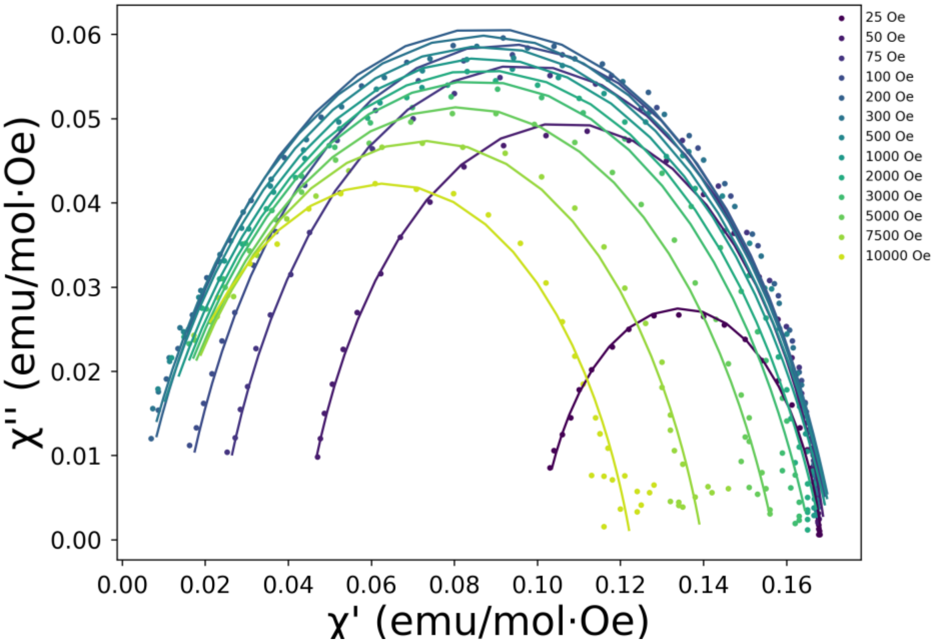

Figure S22. Cole-cole plot for compound **4** acquired at 2.5 K and different fields. Lines are the result of the fit of the data to a Debye function using the CCfit package.

Table S11. Results from the fit of Cole-Cole plots to a Debye function using the CCfit package for **4** at 2.5 K and different applied dc fields.

Field (Oe)	χ_S	χ_t	τ (s)	α	residual
25	1.00E-01	1.69E-01	1.07E-03	1.35E-01	1.04E-05
50	4.36E-02	1.70E-01	1.91E-03	1.52E-01	4.16E-05
75	2.30E-02	1.70E-01	2.44E-03	1.70E-01	6.64E-05
100	1.36E-02	1.71E-01	2.65E-03	1.83E-01	9.84E-05
200	3.15E-03	1.72E-01	2.51E-03	2.06E-01	1.28E-04
300	1.87E-03	1.72E-01	2.19E-03	2.19E-01	1.49E-04
500	2.13E-03	1.72E-01	1.81E-03	2.31E-01	1.59E-04
1000	3.09E-03	1.71E-01	1.41E-03	2.39E-01	1.56E-04
2000	3.98E-03	1.69E-01	1.20E-03	2.42E-01	1.54E-04
3000	4.62E-03	1.66E-01	1.14E-03	2.44E-01	1.20E-04
5000	5.31E-03	1.57E-01	1.06E-03	2.43E-01	9.42E-05
7500	6.55E-03	1.40E-01	7.98E-04	2.13E-01	2.30E-04
10000	4.32E-03	1.22E-01	4.98E-04	2.09E-01	4.01E-04

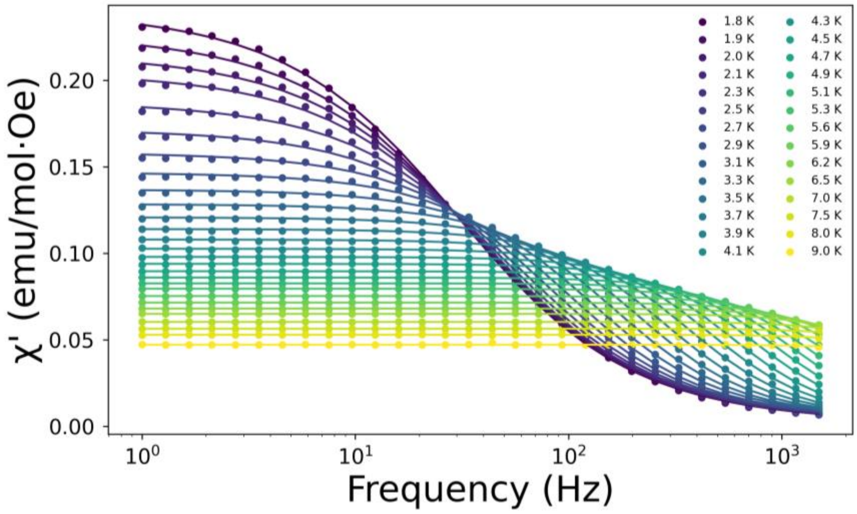

Figure S23. Temperature and frequency dependence of the in phase (χ'_M) component of the ac susceptibility for compound **4** acquired at 200 Oe.

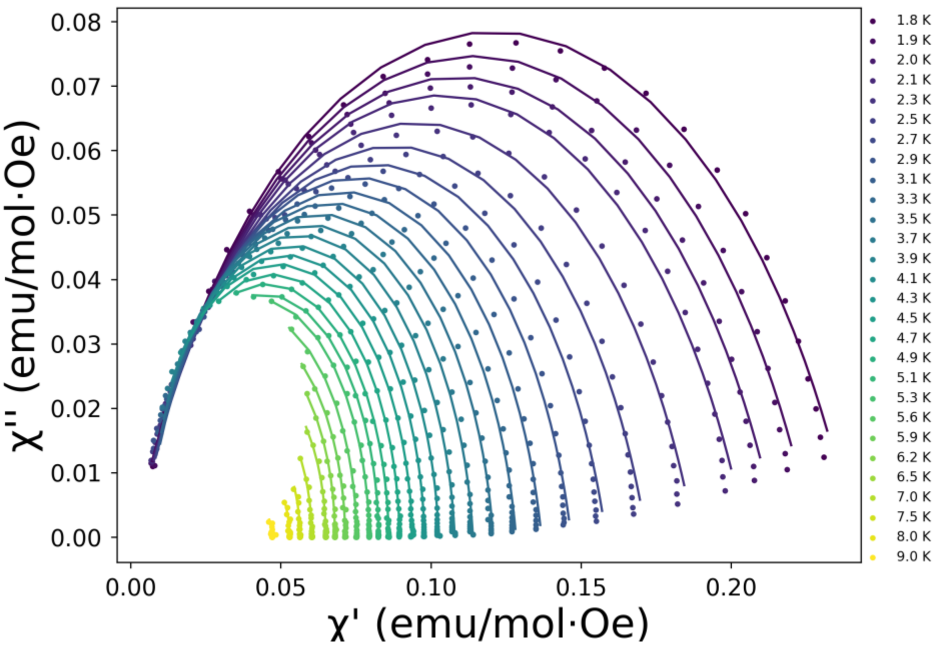

Figure S24. Cole-cole plot for compound **4** acquired at 200 Oe and different temperatures. Lines are the result of the fit of the data to a Debye function using the CCfit package.

Table S12. Results from the fit of Cole-Cole plots to a Debye function using the CCfit package for **3** at 200 Oe applied dc field and different temperatures.

T (K)	χ_S	χ_t	τ (s)	α	residual
1.8	1.24E-03	2.41E-01	5.25E-03	2.62E-01	1.17E-04
1.9	1.56E-03	2.27E-01	4.71E-03	2.56E-01	1.17E-04
2	1.53E-03	2.16E-01	4.22E-03	2.52E-01	1.16E-04
2.1	2.02E-03	2.05E-01	3.80E-03	2.44E-01	1.27E-04
2.3	2.43E-03	1.88E-01	3.12E-03	2.28E-01	1.35E-04
2.5	3.08E-03	1.72E-01	2.51E-03	2.07E-01	1.30E-04
2.7	3.64E-03	1.58E-01	1.99E-03	1.83E-01	1.23E-04
2.9	4.24E-03	1.47E-01	1.57E-03	1.55E-01	1.03E-04
3.1	5.03E-03	1.37E-01	1.23E-03	1.31E-01	9.27E-05
3.3	4.92E-03	1.28E-01	9.50E-04	1.11E-01	6.93E-05
3.5	5.12E-03	1.21E-01	7.35E-04	9.07E-02	4.40E-05
3.7	5.25E-03	1.14E-01	5.69E-04	7.36E-02	3.25E-05
3.9	5.03E-03	1.08E-01	4.39E-04	5.92E-02	2.11E-05
4.1	5.23E-03	1.03E-01	3.47E-04	4.66E-02	1.51E-05
4.31	5.57E-03	9.79E-02	2.77E-04	3.37E-02	7.29E-06
4.5	4.41E-03	9.39E-02	2.17E-04	3.45E-02	6.94E-06
4.7	5.15E-03	8.96E-02	1.76E-04	2.22E-02	3.84E-06
4.9	4.94E-03	8.60E-02	1.43E-04	1.90E-02	3.31E-06
5.1	6.05E-03	8.26E-02	1.18E-04	9.28E-03	2.85E-06
5.3	3.09E-03	7.95E-02	9.25E-05	1.95E-02	1.81E-06
5.6	3.77E-03	7.53E-02	7.13E-05	1.30E-02	2.74E-06
5.9	4.33E-03	7.15E-02	5.46E-05	1.50E-02	2.30E-06
6.2	6.39E-03	6.80E-02	4.57E-05	6.01E-10	1.69E-06
6.5	1.37E-02	6.50E-02	4.08E-05	2.03E-09	2.53E-06
7	1.63E-02	6.03E-02	3.10E-05	2.68E-09	2.14E-06
7.5	2.25E-02	5.64E-02	2.59E-05	3.41E-09	1.66E-06
8	3.24E-02	5.29E-02	3.17E-05	3.05E-09	1.48E-06
9	3.88E-02	4.72E-02	3.93E-05	4.73E-09	3.03E-06

5. Theoretical results

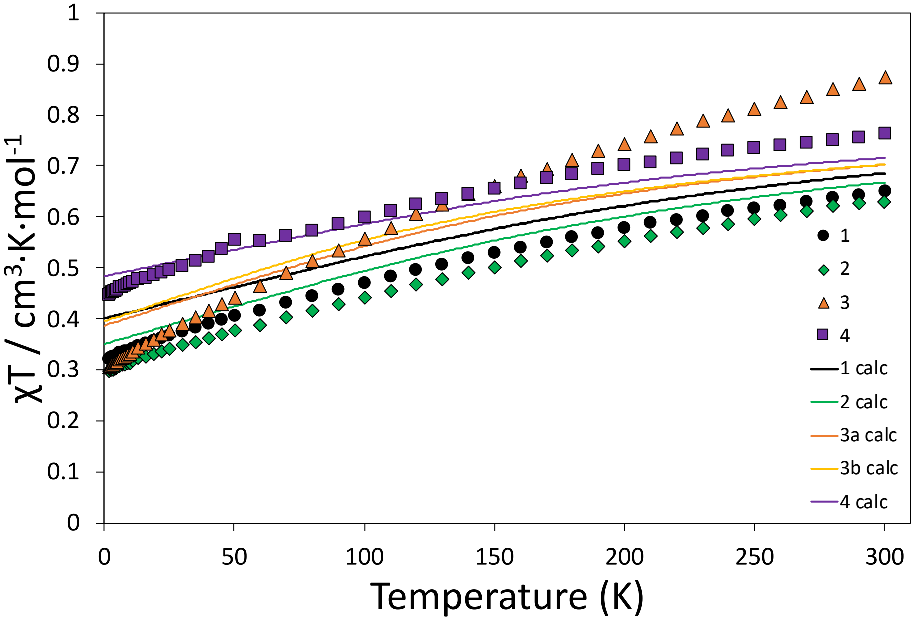

Figure S25. Experimental (symbols) and calculated (lines) $\chi_M T$ vs T plots for compounds **1 - 4**.

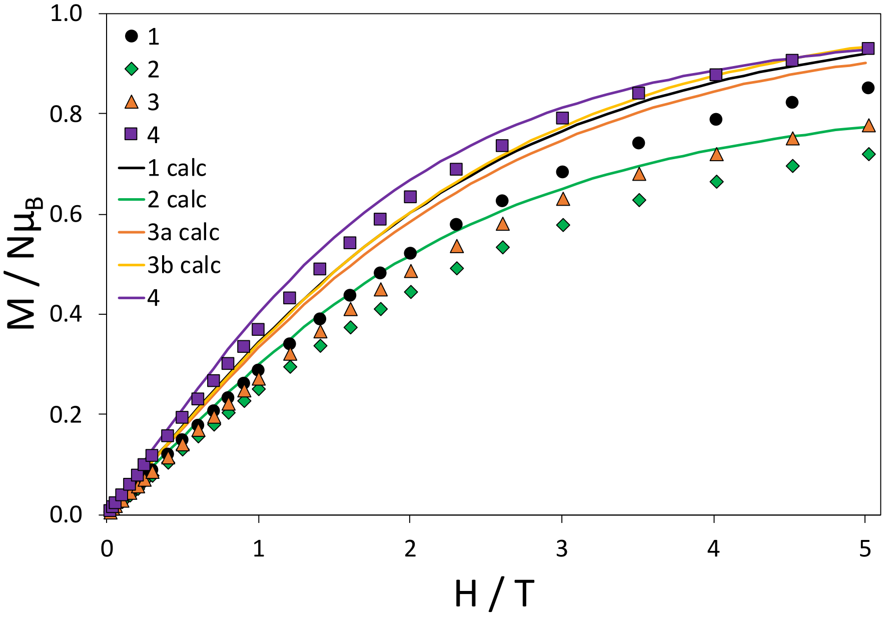

Figure S26. Experimental (symbols) and calculated (lines) M vs H plot for compounds **1** – **4**.

Table S9. Calculated relative Kramers doublet energies (in cm^{-1}) and g tensor components for the ground and first excited doublets at CASSCF-RASSI level for the four studied systems.

		E	g_x	g_y	g_z
1	KD1	0	1.064	1.304	3.160
	KD2	319	2.436	1.934	0.719
	KD3	585	0.224	1.467	2.864
2	KD1	0	0.223	0.374	3.321
	KD2	258	0.150	1.095	3.211
	KD3	644	1.197	1.286	2.803
3[a]	KD1	0	0.850	1.433	3.098
	KD2	282	2.152	1.818	0.033
	KD3	578	0.984	1.353	3.280
3b	KD1	0	0.860	1.837	2.920
	KD2	252	1.025	1.388	1.761
	KD3	572	2.441	1.933	1.167
4	KD1	0	0.092	0.437	3.908
	KD2	325	0.826	1.510	2.827
	KD3	473	0.280	0.706	3.665
